# Skin‐Conformal Ag Flake‐Decorated PEDOT:PSS Sensor Arrays for Spatially Resolved Body Temperature Monitoring

**DOI:** 10.1002/smll.202412675

**Published:** 2025-05-27

**Authors:** Chuljin Hwang, Jun‐Gyu Choi, Changhyun Pang, Min‐Seok Kim, Sungjun Park

**Affiliations:** ^1^ Department of Electrical and Computer Engineering Ajou University Suwon 16499 Republic of Korea; ^2^ School of Chemical Engineering Sungkyunkwan University (SKKU) Suwon 16419 Republic of Korea; ^3^ Mechanical Metrology Group Korea Research Institute of Standards and Science Daejeon 34113 Republic of Korea; ^4^ Department of Intelligence Semiconductor Engineering Ajou University Suwon 16499 Republic of Korea

**Keywords:** polymer‐metal composite, real‐time temperature monitoring, skin‐conformal device, temperature mapping, temperature sensor array

## Abstract

Rapid and spatial temperature measurement on the skin is essential for detecting localized physiological anomalies, such as inflammation or circulatory issues, while providing insights into thermoregulation. Skin‐conformal temperature sensors, with ultra‐flexible designs, enable precise and comfortable measurements, supporting real‐time monitoring, early diagnosis, and effective intervention. However, achieving rapid and spatial skin‐conformal temperature sensor arrays that simultaneously maintain high sensitivity under extreme mechanical stresses remains a significant challenge. This work introduces a skin‐conformal temperature sensor array based on a composite of poly(3,4‐ethylenedioxythiophene):poly(styrene sulfonate) (PEDOT:PSS) and Ag flakes, fabricated on a 2‐µm‐thick parylene‐C substrate. A simple mixing process achieves uniform dispersion of Ag flakes, enhancing electrical conductivity to 2.04 kS cm^−1^. The sensor demonstrates a temperature coefficient of resistance of −2.02%/°C (30–50 °C), a resolution of 0.5 °C, and a rapid response time under 0.41 s per 5 °C change. It endures over 1000 cycles of 200% strain and performs reliably under 3 µm bending radii. Demonstrating high‐resolution sensitivity and spatial temperature mapping through letter pattern recognition, the sensor shows promise for applications in body temperature monitoring, thermal imaging, and early diagnosis of temperature‐related health conditions.

## Graphical abstract

Ultrathin, skin‐conformal sensor arrays based on Ag flake–decorated PEDOT:PSS composites enable high‐resolution, spatially resolved temperature mapping across human skin. The optimized composite formulation exhibits high thermal sensitivity (−2.02% °C⁻¹), mechanical durability, and reliable environmental stability, offering a promising platform for wearable healthcare electronics and future multimodal physiological monitoring.

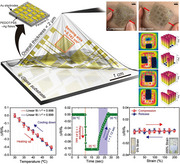

## 1 Introduction

Body temperature is a fundamental physiological parameter that reflects the dynamic balance between heat production and dissipation in the body, serving as a critical indicator of health. It is intricately associated with metabolic activity, as cellular processes such as energy production and enzymatic reactions generate heat, while deviations from normal ranges often signal disruptions in homeostasis.^[^ ^1^, ^2^, ^3^, ^4^ ^]^ Additionally, immune responses, including inflammation and fever, manifest through localized or systemic temperature changes, providing valuable insights into the defense mechanisms of the body against infection or disease.^[^ ^5^, ^6^, ^7^ ^]^ This dual role underscores the importance of monitoring body temperature for diagnosing and managing conditions ranging from metabolic disorders to infectious diseases. While traditional methods, such as mercury thermometers and infrared cameras, are widely used for temperature measurement, they often require over a minute to produce results and cannot provide spatially resolved information about specific affected regions.^[^ ^8^, ^9^ ^]^ Wearable electronics have emerged as a transformative solution, enabling real‐time temperature monitoring and recording, which facilitates the early detection of diseases.^[^ ^10^ ^]^ Considering the narrow physiological temperature range (30–50 °C), recent advancements have focused on thermistors, varying their resistance with temperature changes, directly interfaced with the skin for precise and rapid sensing.^[^ ^11^, ^12^ ^]^

Among flexible temperature sensors, thermistors are mostly suitable for body temperature monitoring due to their high accuracy, cost efficiency, and real‐time measurement.^[^ ^13^, ^14^, ^15^ ^]^ Their critical figure‐of‐merit parameters are sensitivity, response time, resolution, and long‐term stability under repeated thermal cycling. To achieve such performance, temperature‐sensitive materials, including metals,^[^ ^9^, ^13^ ^]^ MXenes,^[^ ^16^, ^17^, ^18^ ^]^ and carbon‐based materials^[^ ^19^, ^20^, ^21^, ^22^ ^]^ (graphene, graphene oxide, and carbon nanotube (CNT)), are often processed into thin films on flexible substrates such as polydimethylsiloxane (PDMS),^[^ ^23^ ^]^ polyethylene terephthalate (PET),^[^ ^24^ ^]^ or polyimide (PI).^[^ ^25^ ^]^ These approaches ensure skin adhesion through introducing ultrathin substrates, bio‐adhesive pastes, and microstructures for mechanical interlocking, but are often constrained by the brittleness of their layered structures and/or fractures caused by bending under skin movement. Their performance becomes unreliable for skin‐conformal sensors, especially under extreme mechanical stresses. As a result, these materials are seldom used alone in skin‐conformal temperature sensors, highlighting the need for polymer‐based composite strategies to address their mechanical limitations.^[^ ^26^, ^27^ ^]^

State‐of‐the‐art research on skin‐conformal temperature sensors has employed conductive polymers, such as poly(3,4‐ethylenedioxythiophene):poly(styrene sulfonate) (PEDOT:PSS), as a temperature‐sensitive composite framework.^[^ ^28^, ^29^ ^]^ Incorporating conductive materials into these composites enhances their electrical performance while achieving mechanical flexibility and compatibility with ultra‐thin substrates. For instance, PEDOT:PSS–graphene oxide composites demonstrated an improved temperature coefficient resistance (TCR) of −1.2%/°C with rapid response times of 3.5 s.^[^ ^30^ ^]^ Similarly, vanadium dioxide nanoparticles are decorated in PEDOT:PSS, followed by spray‐coated onto block‐copolymer fabric.^[^ ^31^ ^]^ The 3 × 3 sensor array functioned as electronic skin, effectively detecting target areas of lower than 10 cm^2^ while demonstrating a TCR of −2.7%/°C and response times as fast as 1 s. Additionally, thermoresponsive hydrogel composites incorporating CNT and PEDOT:PSS exploit bioinspired microstructure substrates for non‐irritating, long‐lasting, and reusable adhesion, which achieved a TCR of −2.6%/°C, albeit with a response time of 139 s.^[^ ^32^ ^]^

However, despite these advancements, achieving rapid and spatial skin‐conformal temperature sensor arrays that simultaneously maintain high sensitivity under extreme mechanical stresses remains a significant challenge. The primary concern arises from deformation‐dependent interparticle distance, attributed to bending or stretching around fine skin wrinkles with bending radii of just a few micrometers.^[^ ^33^ ^]^ Conductive particles smaller than 1 µm are often embedded in temperature‐sensitive polymer matrices, where charge transport primarily occurs through hopping conduction or electron tunneling between adjacent particles.^[^ ^34^, ^35^ ^]^ During mechanical deformation, these conduction mechanisms are affected by increased interparticle distance beyond the effective tunneling range, leading to unstable signal output or reduced measurement consistency. Additionally, while hydrogels provide strain‐reliable and high sensitivity, their volume‐dependent behavior, characterized by swelling and deswelling requires substantial time for equilibrium to be re‐established. This delay slows response times, making hydrogels unsuitable for real‐time monitoring. Spatial monitoring is also challenging due to mechanical strains from fine wrinkles and irregular skin geometries, which disrupt conduction pathways and hinder reliable data collection over broad areas. In arthritis diagnosis, for example, precise mapping of localized inflammation across joints is essential for identifying subtle thermal anomalies and guiding effective treatment,^[^ ^36^ ^]^ highlighting the need for advanced sensor arrays. Therefore, for reliable, real‐time, and long‐term body temperature monitoring, it is imperative to develop sensor arrays that not only conform effectively to the dynamic and spatial contours of the skin but also withstand continuous mechanical deformation, all while maintaining high sensitivity and rapid response.

In this study, we present a 2‐µm‐thick skin‐conformal temperature sensor array, fabricated using a PEDOT:PSS–Ag flake composite to achieve high rapid response and sensitivity under continuous mechanical deformation. The array consists of 16 devices distributed across a 5 × 5 cm^2^ area, enabling spatial and precise temperature measurement under extreme mechanical strains. The sensors demonstrate an exceptional TCR of −2.02%/°C across a temperature range of 30 °C–50 °C with a resolution of 0.5 °C and a rapid response of 0.41 s, which are stable even under humidity variations ranging from 20–60 RH%. Moreover, the sensor array maintains outstanding performance under repetitive mechanical strains (0–200% for 10^3^ cycles in both vertical and horizontal directions) and exhibits mechanical endurance to bending radii below 5 µm without compromising electronic functionalities. Microscopic investigations reveal that Ag flakes larger than 1 µm effectively cover the surface, forming robust and continuous conduction pathways by bridging PEDOT‐rich domains. The capability of this sensor array is further validated through letter mapping based on thermal variations. This finding suggests that our skin‐conformal temperature sensor array holds significant potential for future healthcare systems, enabling real‐time monitoring of body temperature at any desired place.

## 2 Results and Discussion

### 2.1 Design of the Wearable Temperature Sensor Array

**Figure 1:**
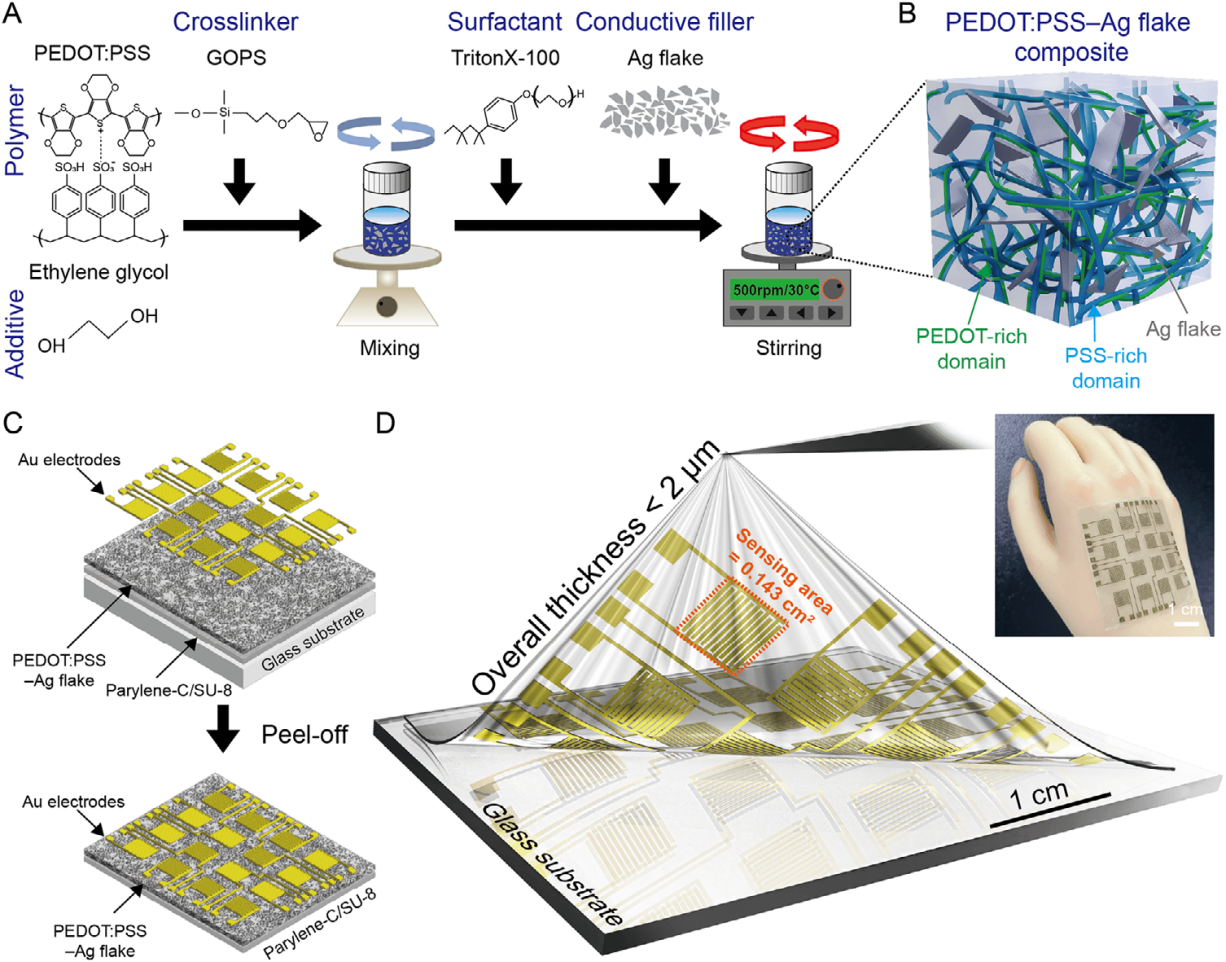
Fabrication and structure of the skin‐conformal temperature sensor array. a) Molecular structures of PEDOT:PSS, GOPS, triton X‐100, and EG, along with the synthesis process of the PEDOT:PSS–Ag flake composite ink. b) Schematic illustration of the molecular morphology of the PEDOT:PSS–Ag flake composite ink, highlighting the PEDOT‐rich and PSS‐rich domains and the embedded Ag flakes. c) Layer‐by‐layer assembly process of the temperature sensor, consisting of PaC, PEDOT:PSS–Ag flake composite, and Au electrodes. The diagram also shows the peel‐off process to obtain the flexible device. d) Fabricated skin‐conformal temperature sensor array on a glass substrate, comprising 16 sensors within a 5 × 5 cm^2^ area. The orange outline marks the effective sensing area of each sensor (0.143 cm^2^) (Top‐right inset: Photograph of the temperature sensor array conformally attached to the back of a hand, demonstrating flexibility and conformal adhesion.).

**Figure** **1**a illustrates the synthetic process of composite ink. First, ethylene glycol (EG) was blended into PEDOT:PSS solution to enhance conductivity. EG effectively reduces the PSS domains and strengthens the interconnection of conductive PEDOT domains, capable of efficient charge transport through the conductive pathway.^[^ ^37^ ^]^ Such conformational changes from coiled to linear structure enable efficient wrapping of PEDOT:PSS around Ag flake in later step.^[^ ^38^ ^]^ Subsequently, to enhance water stability and elasticity, (3‐glycidyloxypropyl) trimethoxy silane (C_9_H_20_O_5_Si) (GOPS) was introduced as a scaffold crosslinker, effectively preventing the dissolution and delamination of PEDOT:PSS films in aqueous solutions.^[^ ^39^ ^]^ However, densified PEDOT:PSS films, arising from chemical crosslinking, impede interchain charge transport and cause a notable reduction in electronic and ionic mobilities.^[^ ^40^ ^]^ Thus, P‐t‐octylophenol (Triton X‐100) and Ag flake were incorporated into the composite ink as non‐ionic surfactant additives and conductive fillers, respectively, to counteract the reduction in conductivity resulting from the addition of GOPS. Triton X‐100 was specifically employed to provide a porosity of PEDOT:PSS film, promoting even distribution of Ag flakes throughout the composite,^[^ ^41^ ^]^ while Ag flakes were strategically incorporated to bridge the gaps between adjacent PEDOT:PSS particles, thereby significantly boosting the conductivity of the PEDOT:PSS–Ag flake composite ink. Figure 1b illustrates a schematic morphology of the prepared PEDOT:PSS–Ag flake composite ink in an aqueous solution. Note that the PEDOT:PSS exhibits a core‐shell structure, with the core primarily composed of a conductive and hydrophobic PEDOT‐rich domain and the shell consisting of an insulating and hydrophilic PSS‐rich domain. Given the hydrophobic nature of Ag flakes, they interact with the conductive PEDOT‐rich domains through preferential affinity between hydrophobic surfaces. By this favorable physical affinity due to reduced interfacial energy, they could promote the formation of conductive pathways for PEDOT:PSS–Ag flake composite ink, even after deposition onto a substrate.

Figure 1c presents a schematic of the complete design of the developed skin‐conformal temperature sensor array, comprising several layers, including PaC, the composite film, and Au electrodes. The detailed fabrication process for the temperature sensor array is illustrated in Figures S1 and S2 (Supporting Information). The devices, prepared on an ultra‐flexible PaC layer, are only 1‐µm‐thick and can withstand high thermal stress up to 290 °C after soft baking to ensure structural integrity. Furthermore, the PaC serves as a free‐standing substrate, enabling easy peel‐off with exceptional flexibility. Figure 1d and Figure S3 (Supporting Information) show the schematic and photograph of the sensor array arranged in a 4 × 4 matrices on a PaC substrate peeled off from a glass substrate, with dimensions of 5 × 5 cm^2^. Each sensor consists of interdigitated electrodes (IDEs) with 12 fingers, a width and length of 250 µm, and an active sensing area of 0.143 cm^2^ (Figure S4, Supporting Information). This IDE structure was carefully chosen to promote uniform current distribution and minimize contact resistance, thereby improving the stability and reproducibility of temperature sensing measurements. Finally, the free‐standing sensor array was conformally attached to the back hand without any wrinkles.

### 2.2 Surface Analysis of PEDOT:PSS–Ag Flake Composite Film

**Figure 2:**
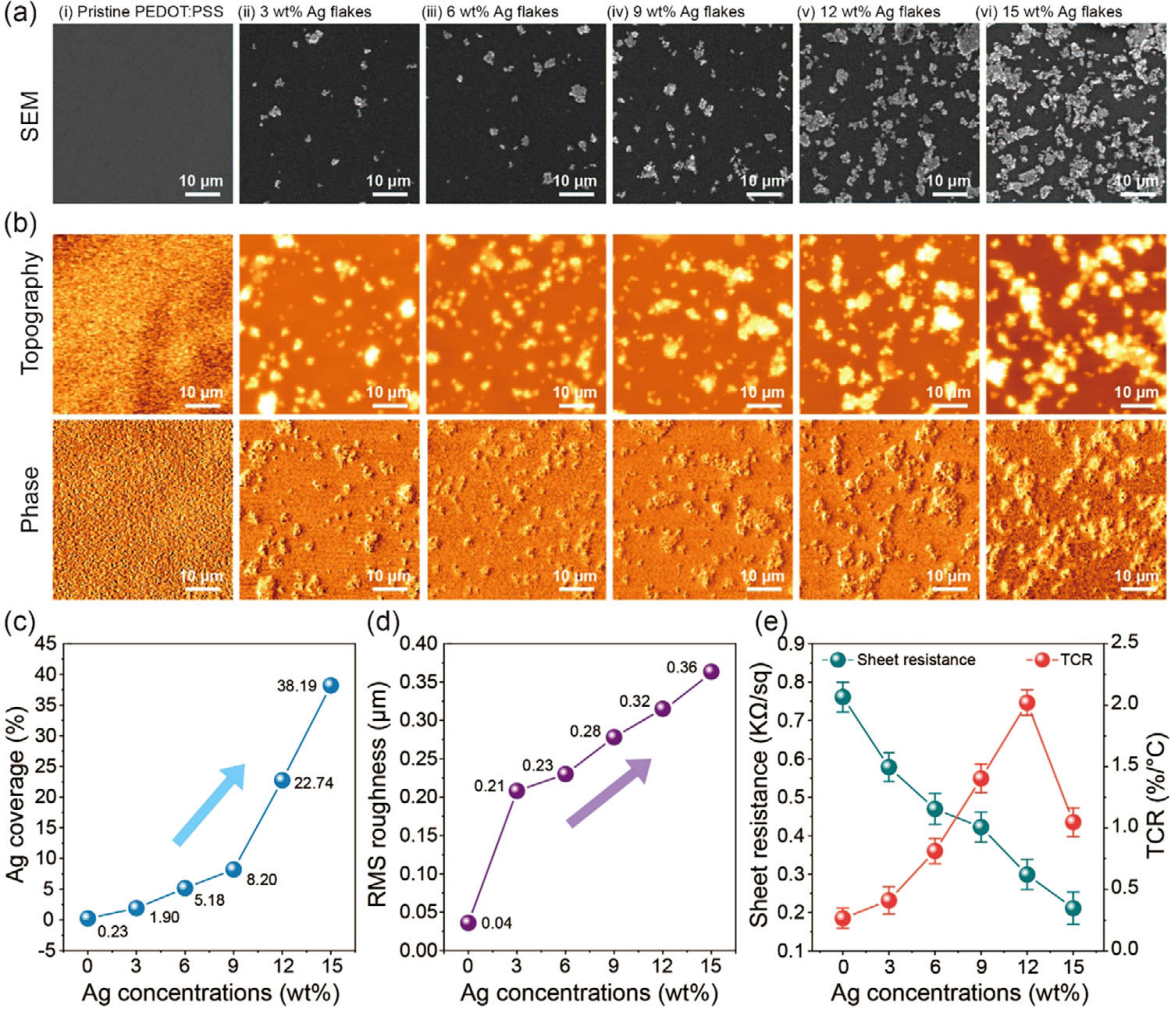
Surface characterization analysis of the temperature sensor. Comparison of surface morphologies at different concentrations of Ag flake using a) scanning electron microscope (SEM) and b) atomic force microscope (AFM) images (top row for topography and bottom row for phase). The samples include (i) pristine PEDOT:PSS and PEDOT:PSS with (ii) 3 wt.%, (iii) 6 wt.%, (iv) 9 wt.%, (v) 12 wt.%, and (vi) 15 wt.% Ag flakes, respectively. c) Changes in Ag coverage and d) root mean square (RMS) roughness on the sensor surface as a function of Ag flake concentration (0–15 wt.%). e) Electrical conductivity for different Ag flake concentrations. The result indicates that the optimal experimental condition for achieving maximum electrical conductivity occurs at an Ag flake concentration of 12 wt.%. Error bars denote standard deviations measured from 20 devices.

For composite thermistor‐based temperature sensors, adjusting the particle concentration is crucial for achieving high conductivity, which is directly linked to optimized sensitivity and a linear response.^[^ ^42^ ^]^ To investigate the electrical and morphological effects of Ag flakes on the composite films, their concentrations varied from 0 to 15 wt.%. **Figure** **2**a,b present the microscopic images of composite films. The field emission scanning electron microscopy (FE‐SEM) images obviously demonstrate that Ag flakes exhibit particle sizes of several micrometers, which grow larger with increasing concentrations. Similarly, the topography and phase diagrams, analyzed by atomic force microscopy (AFM), reveal significant changes in both physical and chemical structures at different Ag flake concentrations.

As the Ag flake concentration increases, the granular morphology significantly intensifies, as reflected in the Ag coverage (Figure 2c), which rises from 0.23% to 38.19%, and the root‐mean‐square (RMS) roughness (Figure 2d), which increases from 0.036 µm to 0.369 µm. An expansion of Ag coverage effectively bridges the conductive PEDOT domains in both longitudinal and transverse directions. This effect is attributed to surface energy‐driven association, namely the preferential affinity between hydrophobic surfaces, leading to consistently dropping sheet resistance. On the other hand, the expansion also results in increased film thickness. The sheet resistance and thickness of composite films as a function of Ag flake concentration are plotted in Figure S5 (Supporting Information), indicating the concentration‐dependent thickness increase due to flake aggregation. Considering that the electrical conductivity is inversely proportional to the product of sheet resistance and film thickness, the excessive film thickness, caused by the significant increase in roughness, can degrade the electrical conductivity. Electrical conductivity was calculated for different Ag flake concentrations, achieving a maximum value of 2.04 kS cm^−1^ at 12 wt.% Ag flake concentration (Figure 2e). The reported sheet resistance values were measured using a four‐point probe method before Au electrode deposition, thus representing the intrinsic electrical properties of the composite films without any influence from the metal contacts. The electrical conductivities of composite films with different Ag flake concentrations are summarized in Table S1 (Supporting Information).

For typical composite thermistors, consisting of an insulating matrix and conductive particles, their thermosensitive behavior is generally described by percolation theory.^[^ ^34^, ^35^ ^]^ This model explains how charge transport transitions from isolated hopping or tunneling at low particle concentrations to continuous network conduction as the percolation threshold is surpassed. In our system, the incorporation of micrometer‐sized Ag flakes led to a steady increase in conductivity with rising filler concentration, suggesting the formation of well‐connected conduction pathways beyond the threshold. Rather than percolation effects alone, the charge transport in this regime was primarily governed by direct physical bridging between adjacent PEDOT‐rich domains. While Ag flake loading beyond 30 wt.% can further enhance conductivity despite the increased film thickness, it leads to a metallic‐dominated temperature response, which is undesirable for thermistor applications. In addition, excessive incorporation of rigid Ag flakes may compromise the mechanical flexibility and conformal adaptability of the composite film, while also making uniform film deposition more difficult. To further improve film uniformity, electrical performance, and mechanical compliance, advanced deposition strategies such as 3D printing^[^ ^43^ ^]^ and PSS‐chain engineering^[^ ^44^ ^]^ may be explored to enable better control over the microstructure of PEDOT:PSS‐based composites.

### 2.3 Electrical Characterization of the PEDOT:PSS–Ag Flake Composite Sensor

**Figure 3:**
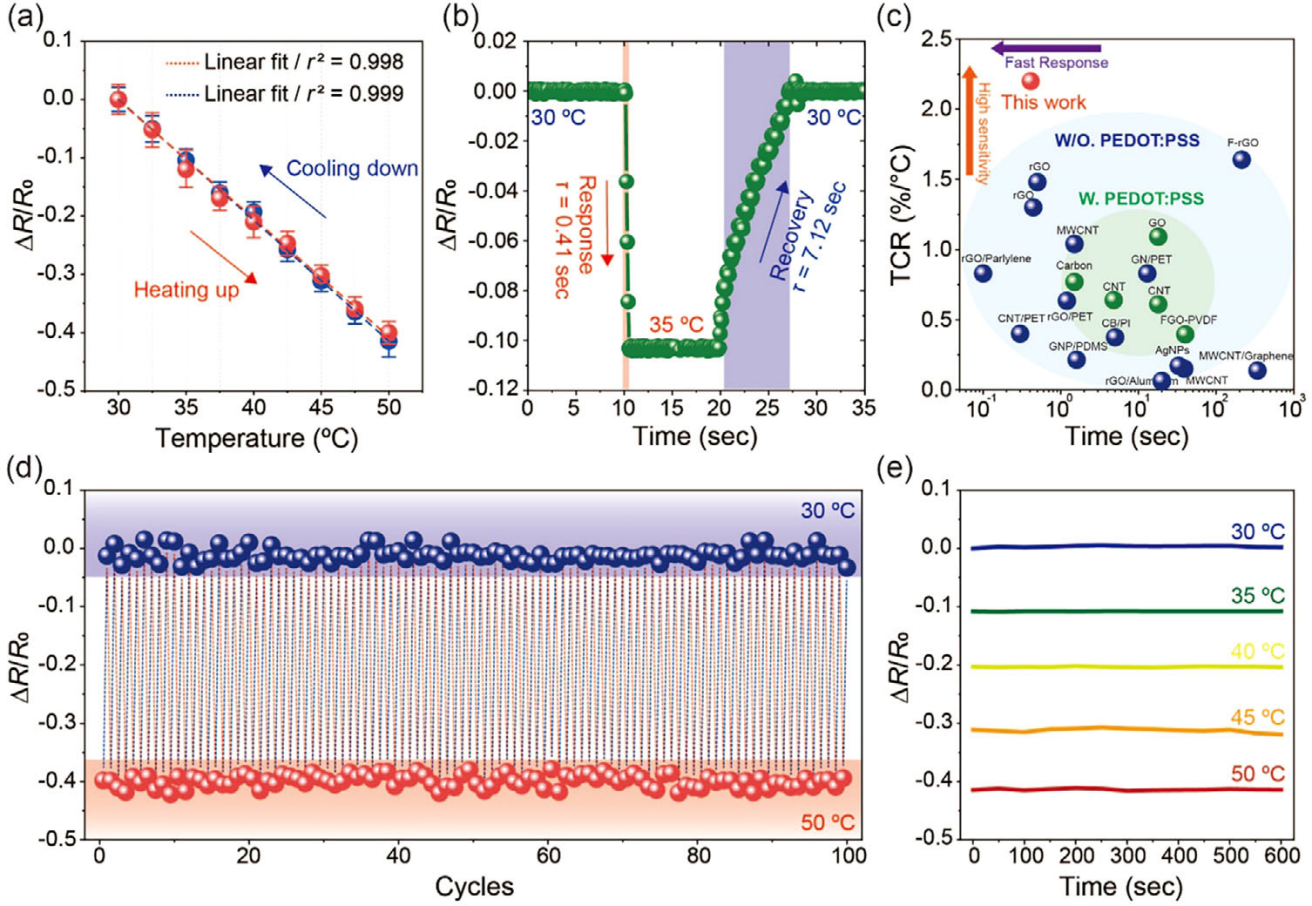
The electrical response of the sensor with reliable and rapid operation. a) Relative resistance changes as a function of temperature, ranging from 30 to 50 °C. The dashed red and blue lines represent linear fitting of the experimental data. Error bars denote standard deviations measured from 30 devices. b) Response and recovery curve during heating and cooling steps, with a temperature change from 30 to 35 °C. c) Overview of the sensing performance, including TCR and response time, compared with various previously reported thermistors. d) Repetitive measurements of relative resistance changes during heating and cooling steps between 30 °C and 50 °C over 100 cycles. e) Continuous measurements of relative resistance over 600 s at 30, 35, 40, 45, and 50 °C, demonstrating stable sensor performance.

After fine optimization of the composite film, we investigated the electrical characteristics of the temperature sensor arrays by measuring relative resistance across a temperature range of 30–50 (C. This specific range was selected to align with the main application of body temperature monitoring. To ensure consistency and eliminate experimental variability, the surface temperatures of each hot plate were pre‐calibrated and verified using infrared thermography. **Figure** **3**a shows a notable decrease in relative resistance of ≈50% for every 10 °C increase. Moreover, the temperature sensor exhibits negligible hysteresis and a superior linear relationship between the change in relative resistance and temperature, as indicated by the high correlation coefficient (*r* ^2^) values of 0.998 during heating and 0.999 during cooling, where a high *r* ^2^ value reflects a strong linear correlation between the experimental data and the theoretical model. Conventional PEDOT:PSS thermistors exhibit negative TCR values due to temperature‐dependent structural deformation between conductive PEDOT‐rich and insulating PSS‐rich domains. This behavior arises from thermally activated hopping conduction across phase‐separated PEDOT‐rich domains, where the insulating PSS matrix acts as a transport barrier.^[^ ^45^, ^46^ ^]^ As the temperature increases, the loosening of hydrogen bonding and structural rearrangement of polymer chains facilitate carrier hopping, resulting in a pronounced negative TCR. However, this hopping‐dominated mechanism often leads to a nonlinear resistance–temperature relationship, which limits sensing accuracy in practical applications. In contrast, micrometer‐sized Ag flakes embedded within the PEDOT:PSS matrix bridged the conductive PEDOT domains more effectively (Figure S6, Supporting Information). These flakes formed robust and continuous conduction pathways that suppressed the variability of hopping distances, enabling more uniform charge transport under thermal stimuli. Notably, resistance changes measured at a resolution of 0.5 °C exhibited an exceptional linear correlation with a high *r* ^2^ value of 0.994 (Figure S7, Supporting Information), indicative of precise measurements of the sensors. As a result, this bridging‐assisted conduction pathway achieved a TCR of –2.02%/°C, approximately eight times higher than that of pristine PEDOT:PSS (–0.26%/°C), indicating significantly enhanced thermal sensitivity and measurement reliability. Note that the observed negative TCR and strong linearity indicate that the sensing behavior originates primarily from the composite, with negligible contribution from the metal electrodes. Furthermore, measurements from 20 individual devices reveal a minimal statistical deviation of −0.088%/°C, reflecting the uniform distribution of the composite ink over the 5 × 5 cm^2^ area and confirming the high reliability of the sensors. The dependence of TCR on Ag flake concentration is plotted in Figure S8 (Supporting Information).

The change in conduction mechanism induced by the addition of ultra‐conductive Ag flakes also contributes to an improved response time. Studying temperature response and recovery times is crucial for understanding the sensitivity of sensors to temperature fluctuations. The response time is defined as the duration required for relative resistance to transition from 90% to 10% of the difference between its initial and final values, while the recovery time is the duration required to transition from 10% to 90% of this difference. As depicted in Figure 3b, the temperature sensor exhibited a significant change in relative resistance when transitioning from 30 to 35 °C, achieving a rapid response time of 0.41 s and a slower recovery time of 7.12 s. This slower recovery is regarded to originating from the thermodynamic asymmetry between heating and cooling. While heating benefits from active energy input via direct contact with a hotplate, cooling relies on passive heat dissipation into a cooler substrate in the absence of an active heat sink. This trend is consistent at different temperatures, specifically 40, 45, and 50 °C, as demonstrated in Figure S9 (Supporting Information). When considering the obtained TCR and response times, the performance of our sensors significantly exceeds that of previously reported counterparts,^[^ ^47^, ^48^, ^49^, ^50^, ^51^, ^52^, ^53^, ^54^, ^55^, ^56^, ^57^, ^58^, ^59^, ^60^, ^61^, ^62^, ^63^, ^64^, ^65^, ^66^ ^]^ as shown in Figure 3c and Table S2 (Supporting Information). These results suggest that our device can respond sensitively to sudden temperature fluctuations, making it highly suitable for real‐time body temperature monitoring, where precise measurements across a wide range and rapid response are essential.

To verify cyclic reliability, a repeatability test was conducted. Figure 3d demonstrates the relative resistance changes during a series of heating and cooling cycles across a broad temperature range, specifically from 30 to 50 °C over 100 cycles. Considering the long‐term operational reliability, the relative resistance changes of the device were also monitored over a period of 7 days (Figure S10, Supporting Information). The results reveal consistent changes in the relative resistance of the sensors, with minimal hysteresis observed between the heating and cooling phases. At the highest and lowest points of the temperature range, slight deviations in resistance change were recorded, with values of −1.04% at 50 °C and −0.64% at 30 °C. Figure 3e illustrates the ability of the sensors to continuously track changes in relative resistance at various temperatures, including 30, 35, 40, 45, and 50 (C, over a prolonged period of 10 min. The sensors exhibit minimal variations in their readings, with a standard error below 0.09%, underscoring their steady and reliable performance across the tested temperature ranges, even without a protective layer in ambient conditions. The effect of humidity on the temperature sensors was also evaluated across a wide relative humidity (RH) range. The sensors were placed in a humidity‐controlled chamber, and relative resistance was measured at varying humidity levels. Figure S11 (Supporting Information) depicts that the relative resistance remained highly stable across RH levels ranging from 20% to 80%, with deviations within ±0.03, which fell within the measurement error margin. This exceptional humidity stability is attributed to the crosslinking effect of GOPS, which forms covalent bonds with the sulfonic acid groups of excess PSS, effectively reducing water uptake and minimizing swelling.^[^ ^42^ ^]^ Despite such excellent stability across a broad humidity range, practical deployment on human skin may involve direct exposure to biological fluids such as sweat, which contain ionic species capable of disrupting the PEDOT:PSS network. To evaluate this, phosphate‐buffered saline (PBS) was used as a model electrolyte, and significant resistance drift was observed in the absence of packaging. This issue was effectively mitigated by introducing a 1 µm‐thick PaC passivation layer on the sensing channel, which successfully suppressed ionic infiltration (Figure S12, Supporting Information). While the mechanical performance of the passivated device under long‐term dynamic deformation has not yet been fully evaluated, the ultrathin geometry of the PaC layer is regarded to help preserve conformal adhesion and flexibility without compromising sensor functionality. Although this passivation strategy was not implemented in the primary sensor design, these findings offer a valuable direction for the future development of chemically resilient, long‐term wearable sensors.

### 2.4 Mechanical Durability of the Sensor Under Tensile Strains

**Figure 4:**
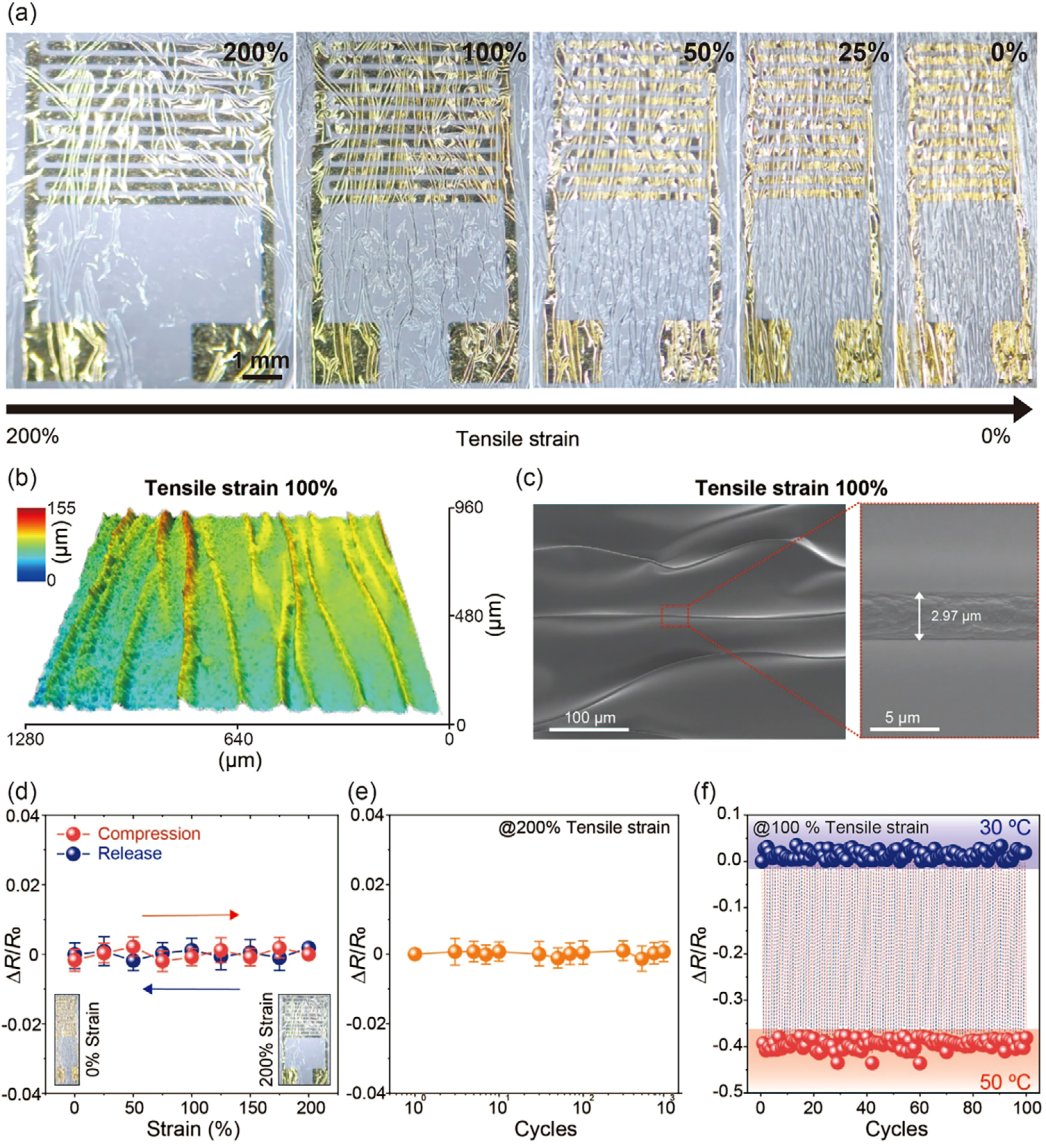
Mechanical durability of the sensor under tensile strains. a) Morphological changes of the sensor under pre‐stretched condition (200% tensile strain) and fully compressed (0% tensile strain) conditions. b) Confocal microscopy image and c) top‐view SEM image of the sensor on PaC substrate under 100% tensile strain. d) Relative resistance changes of the sensor under strains ranging from 0% to 200%. Error bars denote standard deviations measured from 20 devices. e) Relative resistance changes during repeated stretching cycles from 0% to 200% over 10^3^ cycles. f) Relative resistance changes of the sensor during repeated switching between 30 °C and 50 °C for 100 cycles under 100% tensile strain in the horizontal direction.

The transition of the conduction pathway mechanism plays a crucial role in maintaining sensor performance under mechanical strain, as stretching increases the distance between particles with low concentrations or small sizes, potentially disrupting conductivity. To assess durability under mechanical stress, the sensor, fabricated on a 2‐µm‐thick PaC layer on a glass substrate, was peeled off and transferred onto a stretchable elastomer. This setup replicated the conditions of skin attachment, accounting for natural skin tension and movements causing wrinkles. The elastomer, with the sensor attached, underwent controlled compression and release cycles using a strain gauge, as illustrated in Figure S13 (Supporting Information). Initially, the elastomer was pre‐stretched to 200% tensile strain, establishing the initial reference length (*D*_0_). Progressive compression decreased this length to *D*_s_ at full compression (0%), thereby ensuring the reliability and reproducibility of the mechanical durability testing procedure. **Figure** **4**a shows optical images of magnified temperature sensors under mechanical deformation, with strain decreasing from 200% to 0% in 50% compression strain increments, revealing progressively deeper wrinkles as tensile strain decreased in the horizontal direction. Similarly, vertical tensile strain was applied to the sensor, as shown in Figure S14 (Supporting Information). Figure 4b,c presents a 3D profilometry image obtained through confocal microscopy and an SEM image of the composite film under 100% tensile strain in the forward direction, respectively. When subjected to 100% tensile strain, the elastomer formed a unique sinusoidal wrinkled surface with a high aspect ratio, characterized by sharp, multiple bends at the microscale and an exceptionally small bending radius of 3 µm. As demonstrated in our previous research,^[^ ^67^, ^68^ ^]^ the 100% compressive strain effectively mimicked natural skin wrinkles, given that their bending radii can reach down to 5 µm.^[^ ^33^ ^]^

Under significant strain, the durability of the sensor was evaluated by measuring its relative resistance changes in both horizontal and vertical directions. As illustrated in Figure 4d and Figure S15 (Supporting Information), during compression and release cycles between 0% and 200% strain in both directions, the sensor exhibited negligible changes in relative resistance while maintaining stable electrical performance throughout. Conventional thermistors have often exhibited unstable electrical behavior under extreme mechanical deformations, primarily due to their susceptibility to interparticle distances that govern carrier hopping. In contrast, the continuous conduction pathways formed by localized flake interconnection effectively minimized this dependency on hopping distance. Such structural reinforcement is particularly advantageous under realistic skin‐like mechanical conditions, which frequently arise during natural body motion. Impressively, even after 10^3^ cycles of compression and release between these strain levels, as shown in Figure 4e and Figure S16 (Supporting Information), the sensor maintained stable electrical characteristics. The mechanical stability of the sensor was further confirmed during repeated heating and cooling cycles. During dynamic temperature changes under 100% tensile strain, the temperature sensor exhibited excellent electrical performance with minimal baseline drift, as shown in Figure 4f and Figure S17 (Supporting Information). These results demonstrate that the temperature sensor can reliably and stably operate even under severe repetitive mechanical deformation.

### 2.5 Skin‐Conformal Sensor Arrays for Precise and Spatial Temperature Monitoring

**Figure 5:**
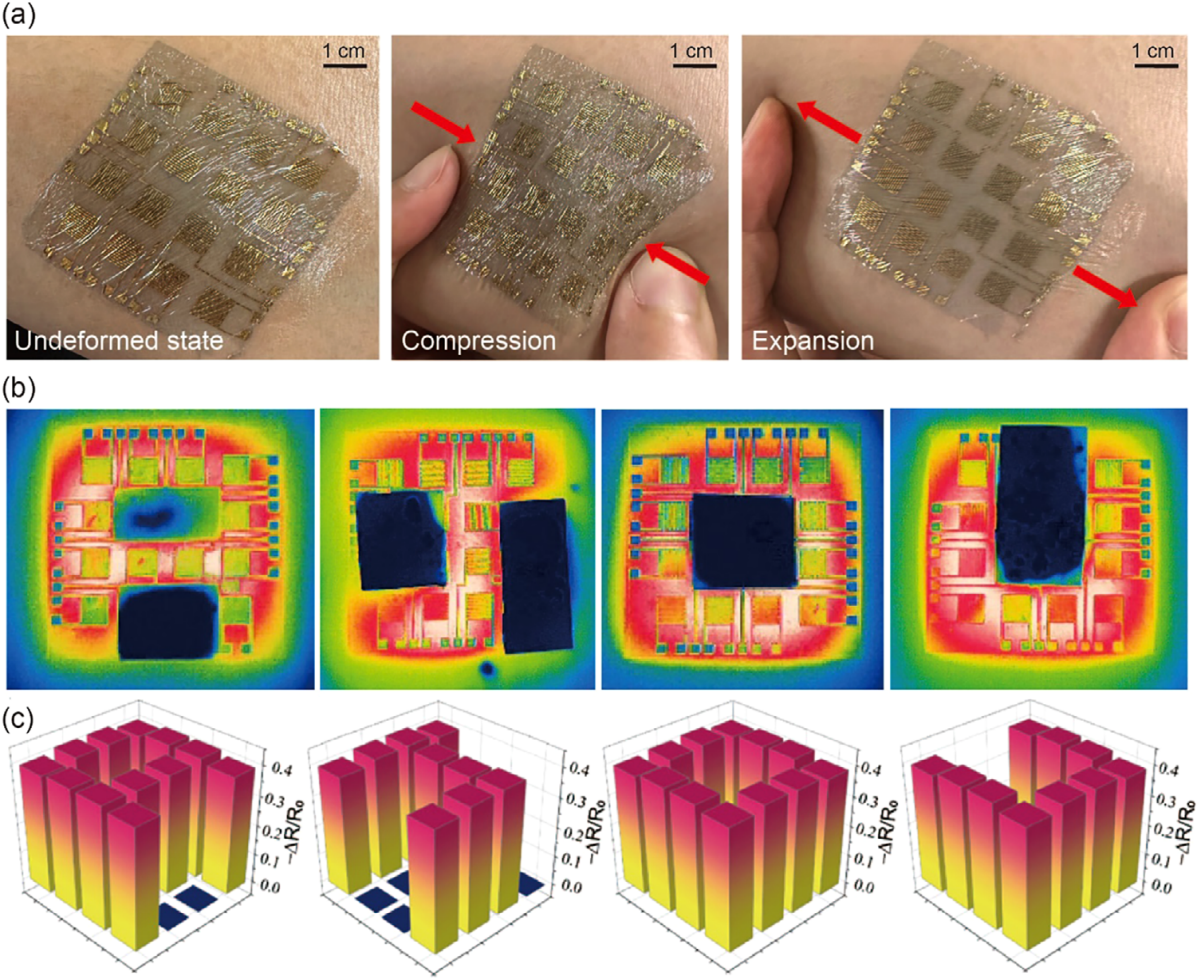
Skin‐conformal sensor array for spatial temperature monitoring. a) Photographic images demonstrating the conformability of the temperature sensor array adhered to the forearm skin surface in different states: undeformed (left), compressed (center), and expanded (right). b) Thermographic images displaying specific heat patterns corresponding to the letters “A,” “J,” “O,” and “U,” generated using an infrared lamp and shading papers, captured by a conventional infrared camera. c) 3D temperature mapping of the relative resistance changes in the sensor array, corresponding to the thermographic images of the letters “A,” “J,” “O,” and “U”.

To ensure the adaptability and effectiveness of the skin‐conformal sensor array, particularly for critical applications such as health monitoring, its conformability was evaluated. The sensor was initially placed on the surface of the forearm, followed by a compression‐expansion test to simulate real‐world conditions. **Figure** **5**a shows a photographic image of the sensor adhered to the forearm, demonstrating its ability to deform naturally and recover without impeding movement. The sensor utilizes a 2‐µm‐thick PaC substrate, known for its stretchability, stability, and biocompatibility, enabling a secure fit on the soft contours of human skin. This minimizes the risk of detachment during movement. Notably, the PaC adheres to the skin through van der Waals forces, eliminating the need for additional adhesives.

To evaluate the mapping ability of the sensor array with temperature distributions, optical and electrical methods were used to analyze heat patterns formed by different shapes, specifically the letters “A”, “J”, “O”, and “U”. Figure 5b presents thermographic image captured using a infrared camera with the aid of an infrared lamp and shading paper for remote heating. Initially, all sensors in the array maintained their baseline resistance, corresponding to a relative resistance change of 0. Upon activation of the infrared lamp, the temperature of the irradiated sensor increased, causing a reduction in resistance, with the relative resistance (*R*/*R*_0_) dropping to ≈−0.4. After the lamp was turned off and the heat dissipated, the relative resistance returned to its baseline level.

Figure 5c illustrates a 3D graphical representation of the temperature distribution, closely aligning with the contours defined by the shading paper and the relative resistance changes induced by the infrared lamp. This high‐resolution capability of the sensor array to map heat distributions is particularly advantageous for detecting subtle temperature variations on human skin, thereby enhancing the reliability of temperature measurements. Unlike traditional methods that focus on single‐point measurements, this sensor array evaluates the average temperature over a defined skin area, allowing accurate and rapid acquisition of temperature profiles across various body regions. Such distributed sensing is not limited to temperature monitoring alone; when integrated with other sensing modalities, it can serve as a foundational component for next‐generation wearable multimodal sensor platforms.^[^ ^69^, ^70^, ^71^ ^]^

## 3 Conclusion

In conclusion, we successfully developed a highly precise and fast‐acting wearable temperature sensor based on a PEDOT:PSS–Ag flake composite. Through SEM and AFM analyses, we established how varying concentrations of Ag flakes affect the composite structure and temperature sensing performance. The sensor exhibited a TCR of 2.02%/°C, superior to existing techniques, along with a prominent resolution of 0.5 °C and stable linear sensitivity across temperatures from 30 to 50 °C. With a rapid response time of 0.41 s, it proved suitable for real‐time human body monitoring. Furthermore, its mechanical reliability was demonstrated through stable performance under repeated deformation cycles. The proof‐of‐concept experiment showcased sub‐1 cm resolution in temperature mapping, exemplified by precise letter pattern recognition through thermal variations. This sensor holds great potential for clinical applications, especially in continuous health monitoring and early detection of abnormal temperature patterns, such as those indicating infections, thereby improving patient outcomes. It also enables ongoing monitoring in daily environments, facilitating diagnosis and evaluation of treatments for temperature‐related conditions.

## 4 Experimental Section

### Material Preparation

PEDOT:PSS (Clevios PH 1000) was purchased from Heraeus (Deutschland). Triton X‐100, EG, and GOPS were purchased from Sigma Aldrich (St. Louis, MO, USA). Ag flakes were purchased from Daejoo Electronic Materials (Gyeonggi‐Do, Korea). SU‐8 and SU‐8 developer were purchased from Kayaku Advanced Materials (Y020100 4000L1PE, USA). Para‐xylylene was purchased from KISCO (311106, Daisan Kasei Co., Ltd.) as a monomer for PaC. All materials were used as received without further purification.

### PEDOT:PSS–Ag Flake Composite Ink Synthesis

PEDOT:PSS, Triton X‐100, EG, GOPS, and Ag flakes were used for the precursor solution. All other reagents were of analytical grade. The composite was mixed at a weight ratio of PEDOT:PSS/TritonX‐100/EG/GOPS = 1/0.03/0.05/0.01. To ensure a uniform distribution of the composite, the solutions were sonicated for 5 min. Subsequently, the mixed precursor solution was stirred for 30 min at room temperature. To further improve the uniform dispersion of Ag flakes and prevent aggregation, a homogenizer was employed during the mixing process.

### Fabrication of Skin‐Conformal Sensor Array

A glass substrate of size 5 × 5 cm^2^ was cleaned, and oxygen plasma was treated at 100 W with 5 sccm for 10 min (Femto science, Korea) to obtain a hydrophilic surface. As a pretreatment process for peeling off the parylene‐c layer, a fluorochemical acrylic polymer EGC‐1700 (3 m Novec Electronic Grade Coating) was dissolved at a volume ratio of 1:10 in HFE‐7100 (3 m Novec Engineered Fluid). Then it was simply deposited through a standard spin coating technique. Then, a PaC was deposited through chemical vapor deposition with a thickness of 1 µm using Lavida‐110H (Femto science, Korea) on the prepared glass substrate. The samples were annealed at 150 °C, lower than the PaC melting temperature (290 °C), for 1 h to form the crystallized PaC layer. After being flattened by spin coating SU‐8 solution, the substrate was treated with oxygen plasma to form interactive bonding sites for PEDOT:PSS–Ag flake composite, while the treatment facilitated epoxide ring opening. Subsequently, the PEDOT:PSS–Ag flake composite inks were spin‐coated at 1500 rpm for 60 s and annealed at 140 °C for 0.5 h. The final device was completed by depositing gold electrodes (50 nm‐thick) using conventional thermal evaporation.

### Surface Characterization

The morphologies of the PEDOT:PSS–Ag flake composite film were conducted by a field emission scanning electron microscope (FE‐SEM I, S‐4800, Hitachi) and atomic force microscopy (AFM; XE‐100, Park Systems, Korea). Electrical characterization was conducted using a Keithley 2400 source meter (Keithley, Cleveland, USA) at ambient conditions with a DC sweep from −1 to 1 V and recorded with the corresponding data acquisition system. The film thickness was measured using an Alpha‐step (KLA‐Tencor D‐300).

### Electrical Characterization of Skin‐Conformal Temperature Sensor Arrays

Electrical conductivities of PEDOT: PSS–Ag flake composite films were calculated via the 4‐point probe method in each condition using the following Equation (1):

(1) $$\sigma =\frac{1}{R\;\times \;d}\;\left(S/\mathrm{cm}\right)$$

where σ, R , and d represent the electrical conductivity, sheet resistance, and thickness of the composite film, respectively. The thermal sensing capability of the temperature sensor arrays strongly depends on TCR, which quantifies the sensitivity to changes in temperature. Thus, a larger TCR value denotes that the material is more sensitive to changes in temperature, and the following calculation formula was used in Equation (2):

(2) $$TCR=\frac{R-R_{0}}{R_{0}}\times \frac{1}{\Delta T}\times 100\;\left(\%{/}^{\circ }C\right)$$

*R* denotes the resistance at the temperature, while *R*_0_ denotes the starting resistance, and Δ*T* describes the temperature change. To establish electrical contact for skin‐mounted devices, Au wiring patterns (100 nm thick) were thermally evaporated onto a 12.5‐µm‐thick polyimide substrate using a shadow mask. The sensing device was then bonded to these Au lines using conductive 3 m adhesive tape, ensuring a stable and low‐resistance interface. Finally, the Au lines were connected to a Keithley 2400 sourcemeter (Keithley, Cleveland, USA) via alligator clips, allowing for accurate and real‐time temperature measurements.

### Statistical Analysis

As described in the main text, the figure‐of‐merit parameters, including sheet resistance, TCR, thickness, and relative resistance changes, were statistically analyzed by measuring more than 20 spatially distributed films or devices for each dataset.

## Conflict of Interest

The authors declare no conflict of interest.

## Supporting information



Supporting Information

## Data Availability

The data that support the findings of this study are available from the corresponding author upon reasonable request.

## References

[1] H. Jeong , J. A. Rogers , S. Xu , Sci. Adv. 2020, 6, abd4794. 10.1126/sciadv.abd4794 PMC7467694 32917604

[2] S. Mirjalali , S. Peng , Z. Fang , C.‐H. Wang , S. Wu , Adv. Mater. Technol. 2022, 7, 2100545. 34901382 10.1002/admt.202100545 PMC8646515

[3] H. K. Tripathy , S. Mishra , S. Suman , A. Nayyar , K. S. Sahoo , Computing 2022, 104, 1233.

[4] G. Quer , J. M. Radin , M. Gadaleta , K. Baca‐Motes , L. Ariniello , E. Ramos , V. Kheterpal , E. J. Topol , S. R. Steinhubl , Nat. Med. 2021, 27, 73. 33122860 10.1038/s41591-020-1123-x

[5] Y. Lu , Y. Fujita , S. Honda , S.‐H. Yang , Y. Xuan , K. Xu , T. Arie , S. Akita , K. Takei , Adv. Healthc. Mater. 2021, 10, 2100103. 10.1002/adhm.202100103 33955182

[6] J. Huan , J. S. Bernstein , P. Difuntorum , N. V. R. Masna , N. Gravenstein , S. Bhunia , S. Mandal , IEEE Sens. J. 2022, 22, 1670.

[7] R. C. Webb , A. P. Bonifas , A. Behnaz , Y. Zhang , K. J. Yu , H. Cheng , M. Shi , Z. Bian , Z. Liu , Y.‐S. Kim , W.‐H. Yeo , J. S. Park , J. Song , Y. Li , Y. Huang , A. M. Gorbach , J. A. Rogers , Nat. Mater. 2013, 12, 938. 24037122 10.1038/nmat3755 PMC3825211

[8] W. Fan , T. Liu , F. Wu , S. Wang , S. Ge , Y. Li , J. Liu , H. Ye , R. Lei , C. Wang , Q. Che , Y. Li , ACS Nano 2023, 17, 21073. 37874666 10.1021/acsnano.3c04246 PMC10655239

[9] M. Sang , K. Kang , Y. Zhang , H. Zhang , K. Kim , M. Cho , J. Shin , J.‐H. Hong , T. Kim , S. K. Lee , W.‐H. Yeo , J. W. Lee , T. Lee , B. Xu , K. J. Yu , Adv. Mater. 2022, 34, 2105865. 10.1002/adma.202105865 34750868

[10] N. Verma , I. Haji‐Abolhassani , S. Ganesh , J. Vera‐Aguilera , J. Paludo , R. Heitz , S. N. Markovic , K. Kulig , A. Ghoreyshi , IEEE J. Transl. Eng. Health Med. 2021, 9, 1. 10.1109/JTEHM.2021.3098127 PMC8577572 34765323

[11] S. Nakata , T. Arie , S. Akita , K. Takei , ACS Sens. 2017, 2, 443. 28723207 10.1021/acssensors.7b00047

[12] M. Huang , T. Tamura , T. Yoshimura , T. Tsuchikawa , S. Kanaya , presented at 38th Annual Int. Conf. of the IEEE Engineering in Medicine and Biology Society (EMBC) , Orlando, FL, USA, August 2016. 10.1109/EMBC.2016.7590669 28268308

[13] Z. Peng , W. Guo , T. Liu , X. Wang , D. Shen , Y. Zhu , X. Zhou , J. Yan , H. Zhang , ACS Appl. Mater. Interfaces 2024, 16, 10496. 38377380 10.1021/acsami.3c15995

[14] Y.‐J. Kim , T.‐S. D. Le , H. K. Nam , D. Yang , B. Kim , CIRP Ann. 2021, 70, 443.

[15] A. M. Khalaf , J. L. Ramírez , S. A. Mohamed , H. H. Issa , Flex. Print. Electron. 2022, 7, 045012.

[16] J. Pang , S. Peng , C. Hou , X. Wang , T. Wang , Y. Cao , W. Zhou , D. Sun , K. Wang , M. H. Rümmeli , G. Cuniberti , H. Liu , Nano Res. 2023, 16, 5767.

[17] S. Hao , Q. Fu , L. Meng , F. Xu , J. Yang , Nat. Commun. 2022, 13, 6472. 36309511 10.1038/s41467-022-34168-x PMC9617538

[18] H. Ding , Y. Wang , X. Shen , ACS Appl. Polym. Mater. 2023, 5, 4678. 10.1021/acsapm.3c00500 37552710

[19] J. Wu , X. Yang , H. Ding , Y. Wei , Z. Wu , K. Tao , B.‐R. Yang , C. Liu , X. Wang , S. Feng , X. Xie , Adv. Electron. Mater. 2020, 6, 2000451.

[20] M. Umar , F. S. Irani , S. S. Mirbakht , M. K. Yapici , Adv. Electron. Mater. 2024, 10, 2300723.

[21] S. Behera , Nanotechnology 2024, 35, 505710. 10.1088/1361-6528/ad7f5e 39321822

[22] J. Wu , W. Huang , Y. Liang , Z. Wu , B. Zhong , Z. Zhou , J. Ye , K. Tao , Y. Zhou , X. Xie , Adv. Electron. Mater. 2021, 7, 2001084.

[23] M. Wagih , J. Shi , M. Li , A. Komolafe , T. Whittaker , J. Schneider , S. Kumar , W. Whittow , S. Beeby , Nat. Commun. 2024, 15, 452. 38199999 10.1038/s41467-024-44735-z PMC10781794

[24] C. Okutani , T. Yokota , T. Someya , Adv. Sci. 2022, 9, 2202312. 10.1002/advs.202202312 PMC9596841 36057993

[25] Y. Houeix , S. Habboush , S. Gomez‐Gijon , N. Rodriguez , F. J. Romero , A. Rivadeneyra , Flex. Print. Electron. 2024, 9, 035010.

[26] A. Kobyliukh , Y. Mamunya , M. Godzierz , K. Olszowska , S. Pusz , M. Musioł , U. Szeluga , Adv. Eng. Mater. 2024, 26, 2401558.

[27] L. Xu , L. Li , L. Tang , Y. Zeng , G. Chen , C. Shao , C. Wu , G. He , Q. Chen , G. Fang , D. Sun , Z. Hai , ACS Appl. Mater. Interfaces 2023, 15, 9996. 36780511 10.1021/acsami.2c20927

[28] D. Jucius , R. Gudaitis , A. Lazauskas , V. Grigaliūnas , Polymers 2021, 13, 3519. 34685277 10.3390/polym13203519 PMC8541043

[29] P. Saxena , P. Shukla , J. Electrochem. Soc. 2024, 171, 047504.

[30] C. Zhou , N. Tang , X. Zhang , Y. Fang , Y. Jiang , H. Zhang , X. Duan , Front. Chem. 2020, 8, 194. 32266213 10.3389/fchem.2020.00194 PMC7098694

[31] Y. Wang , Y. Hong , X. Hu , Y. Ye , P. Wang , J. Luo , A. Yin , Z. Ren , H. Liu , X. Qi , J. Liu , S. He , S. Yu , J. Wei , Adv. Mater. Technol. 2023, 8, 2300898.

[32] J. H. Oh , S. Y. Hong , H. Park , S. W. Jin , Y. R. Jeong , S. Y. Oh , J. Yun , H. Lee , J. W. Kim , J. S. Ha , ACS Appl. Mater. Interfaces 2018, 10, 7263. 29400434 10.1021/acsami.7b17727

[33] S. Weiss , J. Moulin , P. Chandran , G. Zoss , P. Gotardo , D. Bradley , Comput. Graph. Forum 2023, 42, 14904.

[34] P. Fourmont , Y. Bai , F.‐X. Fortier , S. G. Cloutier , ACS Appl. Electron. Mater. 2022, 4, 5905.

[35] F. Lai , L. Zhao , J. Zou , P. Zhang , React. Funct. Polym. 2020, 151, 104562.

[36] Y. Nam , D. Shin , J.‐G. Choi , I. Lee , S. Moon , Y. Yun , W.‐J. Lee , I. Park , S. Park , J. Lee , Small Methods 2024, 8, 2301735. 10.1002/smtd.202301735 38529746

[37] Q. Wei , M. Mukaida , K. Kirihara , Y. Naitoh , T. Ishida , Materials 2015, 8, 732. 28787968 10.3390/ma8020732 PMC5455279

[38] Q. Wei , M. Mukaida , Y. Naitoh , T. Ishida , Adv. Mater. 2013, 25, 2831. 23606373 10.1002/adma.201205158

[39] J. Rivnay , P. Leleux , M. Ferro , M. Sessolo , A. Williamson , D. A. Koutsouras , D. Khodagholy , M. Ramuz , X. Strakosas , R. M. Owens , C. Benar , J.‐M. Badier , C. Bernard , G. G. Malliaras , Sci. Adv. 2015, 1, 1400251. 10.1126/sciadv.1400251 PMC4640642 26601178

[40] A. Håkansson , S. Han , S. Wang , J. Lu , S. Braun , M. Fahlman , M. Berggren , X. Crispin , S. Fabiano , J. Polym. Sci. Part B Polym. Phys. 2017, 55, 814.

[41] R. Luo , H. Li , B. Du , S. Zhou , Y. Zhu , Org. Electron. 2020, 76, 105451.

[42] Y.‐F. Wang , T. Sekine , Y. Takeda , K. Yokosawa , H. Matsui , D. Kumaki , T. Shiba , T. Nishikawa , S. Tokito , Sci. Rep. 2020, 10, 2467. 32051489 10.1038/s41598-020-59432-2 PMC7016104

[43] J. Yu , R. Wan , F. Tian , J. Cao , W. Wang , Q. Liu , H. Yang , J. Liu , X. Liu , T. Lin , J. Xu , B. Lu , Small 2024, 20, 2308778. 10.1002/smll.202308778 38063822

[44] J. Yu , F. Tian , W. Wang , R. Wan , J. Cao , C. Chen , D. Zhao , J. Liu , J. Zhong , F. Wang , Q. Liu , J. Xu , B. Lu , Chem. Mater. 2023, 35, 5936.

[45] J. Li , H. Nie , G. Zhou , Y. Hong , W. Meng , Y. Zhu , Q. Huang , Sens. Actuators Phys. 2023, 363, 114706.

[46] Z. Fan , J. Ouyang , Adv. Electron. Mater. 2019, 5, 1800769.

[47] Q. Liu , H. Tai , Z. Yuan , Y. Zhou , Y. Su , Y. Jiang , Adv. Mater. Technol. 2019, 4, 1800594.

[48] Z. Cui , F. R. Poblete , Y. Zhu , ACS Appl. Mater. Interfaces 2019, 11, 17836. 30985098 10.1021/acsami.9b04045

[49] D. Barmpakos , V. Belessi , R. Schelwald , G. Kaltsas , Nanomaterials 2021, 11, 2025. 34443855 10.3390/nano11082025 PMC8399739

[50] D. Kong , L. T. Le , Y. Li , J. L. Zunino , W. Lee , Langmuir 2012, 28, 13467. 22924965 10.1021/la301775d

[51] B. A. Kuzubasoglu , E. Sayar , C. Cochrane , V. Koncar , S. K. Bahadir , J. Mater. Sci. Mater. Electron. 2021, 32, 4784.

[52] B. A. Kuzubasoglu , E. Sayar , S. K. Bahadir , IEEE Sens. J. 2021, 21, 13090.

[53] M. Hilal , J. I. Han , IEEE Sens. J. 2020, 20, 5146.

[54] S. Ali , S. Khan , A. Bermak , IEEE Access 2019, 7, 163981.

[55] J. Zikulnig , M. Khalifa , L. Rauter , H. Lammer , J. Kosel , Chemosensors 2021, 9, 95.

[56] J. Huang , X. Yang , S.‐C. Her , Y.‐M. Liang , Sensors 2019, 19, 317.30646618

[57] P. Sehrawat , Abid, S. S. I. , P. Mishra , Sens. Actuators, B 2018, 258, 424.

[58] J. Yang , D. Wei , L. Tang , X. Song , W. Luo , J. Chu , T. Gao , H. Shi , C. Du , RSC Adv. 2015, 5, 25609.

[59] V. S. Turkani , D. Maddipatla , B. B. Narakathu , B. J. Bazuin , M. Z. Atashbar , Sens. Actuators Phys. 2018, 279, 1.

[60] L. Wu , J. Qian , J. Peng , K. Wang , Z. Liu , T. Ma , Y. Zhou , G. Wang , S. Ye , J. Mater. Sci. Mater. Electron. 2019, 30, 9593.

[61] W. Honda , S. Harada , T. Arie , S. Akita , K. Takei , Adv. Funct. Mater. 2014, 24, 3299.

[62] T. Vuorinen , J. Niittynen , T. Kankkunen , T. M. Kraft , M. Mäntysalo , Sci. Rep. 2016, 6, 35289. 27752050 10.1038/srep35289 PMC5082757

[63] R. Polanský , R. Soukup , J. Řeboun , J. Kalčík , D. Moravcová , L. Kupka , M. Švantner , P. Honnerová , A. Hamáček , Sens. Actuators Phys. 2017, 265, 111.

[64] B. B. Maskey , K. Shrestha , J. Sun , H. Park , J. Park , S. Parajuli , S. Shrestha , Y. Jung , S. Ramasundaram , G. R. Koirala , G. Cho , RSC Adv. 2020, 10, 12407. 35497615 10.1039/d0ra00554a PMC9050635

[65] O. Ozioko , Y. Kumaresan , R. Dahiya , 2020 IEEE Int. Conf. Flex. Printable Sens. Syst. FLEPS 2020, 1, 10.1109/FLEPS49123.2020.9239431.

[66] C. Bali , A. Brandlmaier , A. Ganster , O. Raab , J. Zapf , A. Hübler , Mater. Today Proc. 2016, 3, 739.

[67] I. Lee , J. H. Kim , Y. Kim , D. Shin , H. Lee , J. Won , K. Kang , J.‐G. Choi , M.‐H. Yoon , S. Park , Adv. Mater. 2025, 37, 2410444. 10.1002/adma.202410444 39491808

[68] S. Park , S. W. Heo , W. Lee , D. Inoue , Z. Jiang , K. Yu , H. Jinno , D. Hashizume , M. Sekino , T. Yokota , K. Fukuda , K. Tajima , T. Someya , Nature 2018, 561, 516. 30258137 10.1038/s41586-018-0536-x

[69] J. Qiu , X. Yu , X. Wu , Z. Wu , Y. Song , Q. Zheng , G. Shan , H. Ye , M. Du , Small 2023, 19, 2205324. 10.1002/smll.202205324 36634985

[70] H. Cho , I. Lee , J. Jang , J.‐H. Kim , H. Lee , S. Park , G. Wang , Nat. Electron. 2023, 6, 619.

[71] D.‐H. Kang , J.‐G. Choi , W.‐J. Lee , D. Heo , S. Wang , S. Park , M.‐H. Yoon , APL Bioeng. 2023, 7, 026102. 37056513 10.1063/5.0138861 PMC10089684

