# Supplementary material for: Skin‐Conformal Ag Flake‐Decorated PEDOT:PSS Sensor Arrays for Spatially Resolved Body Temperature Monitoring
Source: Small. 2025 May 27;21(32):2412675. doi: 10.1002/smll.202412675 (PMC12366251; doi:10.1002/smll.202412675)
Supplement: Supplementary file 1 — Supporting Information [file SMLL-21-2412675-s001.docx]

**Skin-Conformal Ag Flake-Decorated PEDOT:PSS Sensor Arrays for Spatially Resolved Body Temperature Monitoring**

Chuljin Hwang^a†^, Jun-Gyu Choi^a†^, Changhyun Pang^b^*, Min-Seok Kim^c^**, and Sungjun Park^a,d^***

*^a^Department of Electrical and Computer Engineering, Ajou University, Suwon 16499, Republic of Korea*

*^b^School of Chemical Engineering, Sungkyunkwan University (SKKU), Suwon, 16419, Republic of Korea*

*^c^Mechanical Metrology Group, Korea Research Institute of Standards and Science, Daejeon, 34113, Republic of Korea*

*^d^Department of Intelligence Semiconductor Engineering, Ajou University, Suwon 16499, Republic of Korea*

*Corresponding Author: Prof. Changhyun Park (chpang@skku.edu)

**Corresponding Author: Dr. Min-Seok Kim (minsk@kriss.re.kr)

***Corresponding Author: Prof. Sungjun Park (sj0223park@ajou.ac.kr)


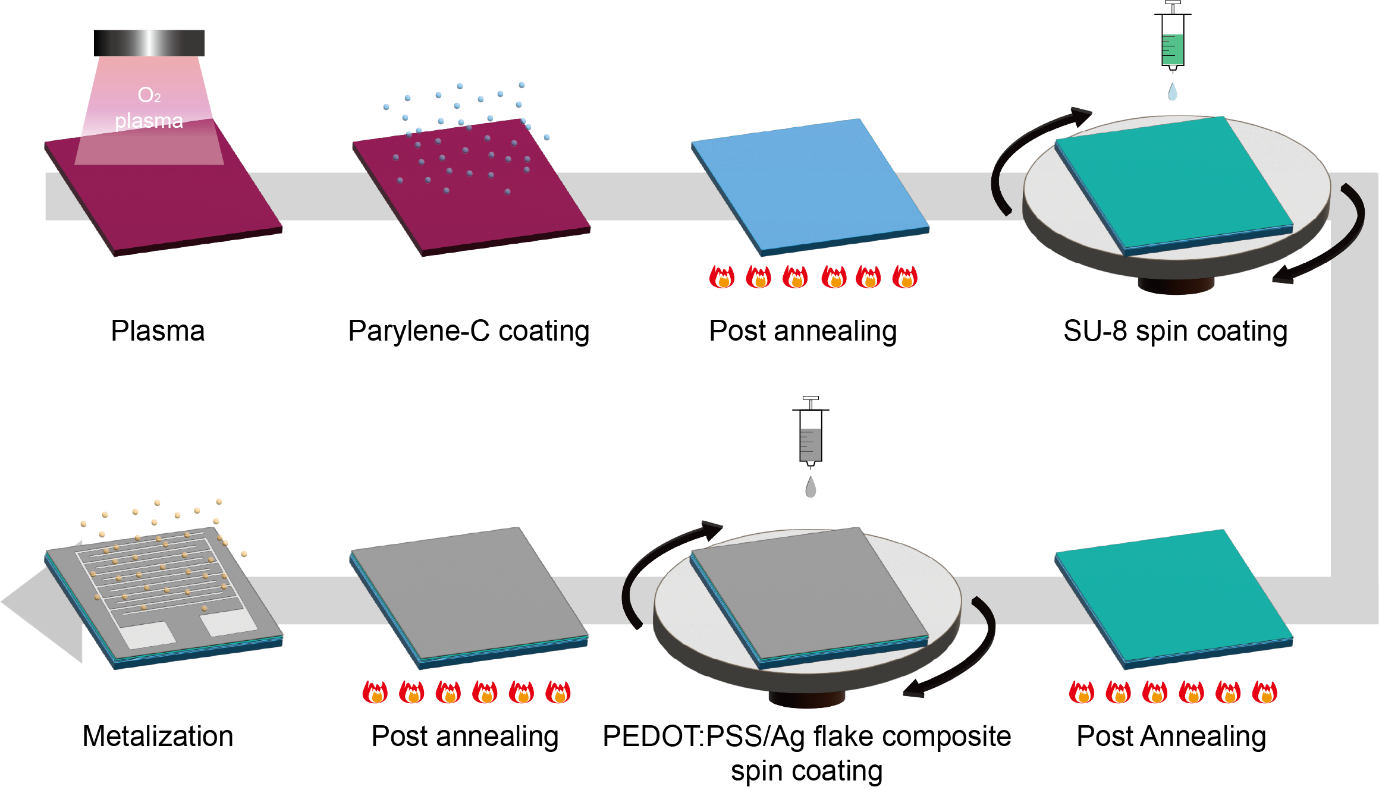


**Figure S1.** Fabrication process of wearable temperature sensor array based on PEDOT:PSS–Ag flake composite.


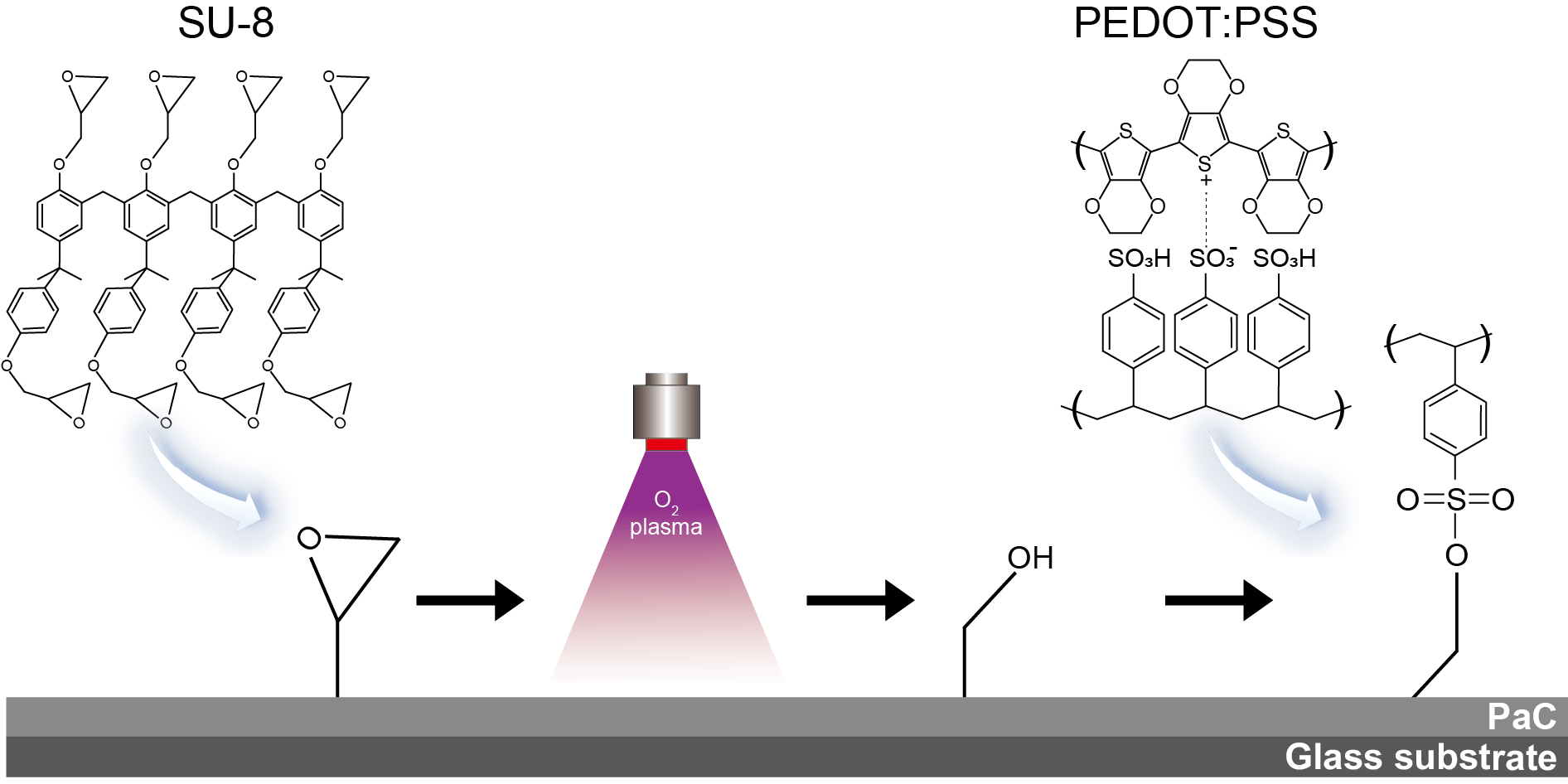


**Figure S2.** Schematic of detailed coating processes, illustrating covalent bonding between activated epoxide groups on SU-8 and sulfonic acid groups of PSS in PEDOT:PSS.


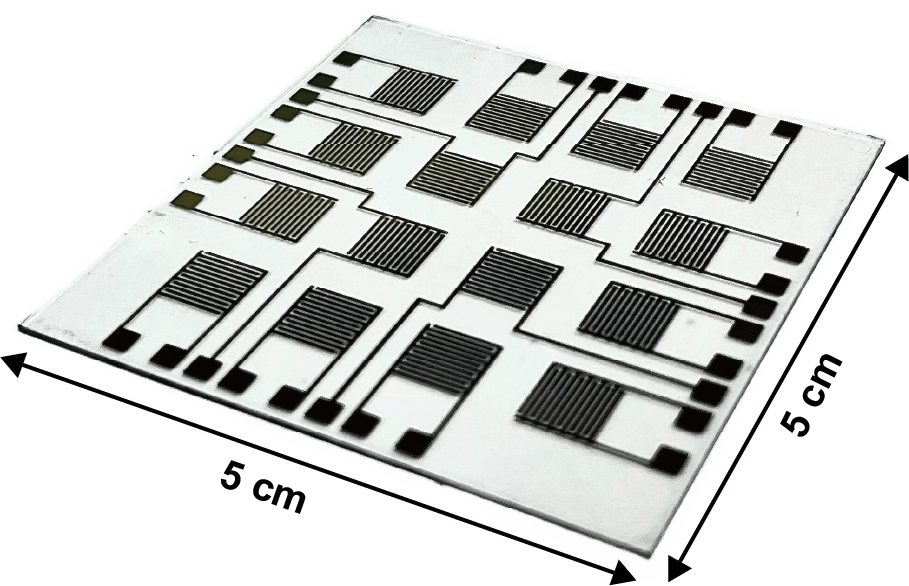


**Figure S3.** Photograph image of fully fabricated wearable temperature sensor array on the glass substrate.


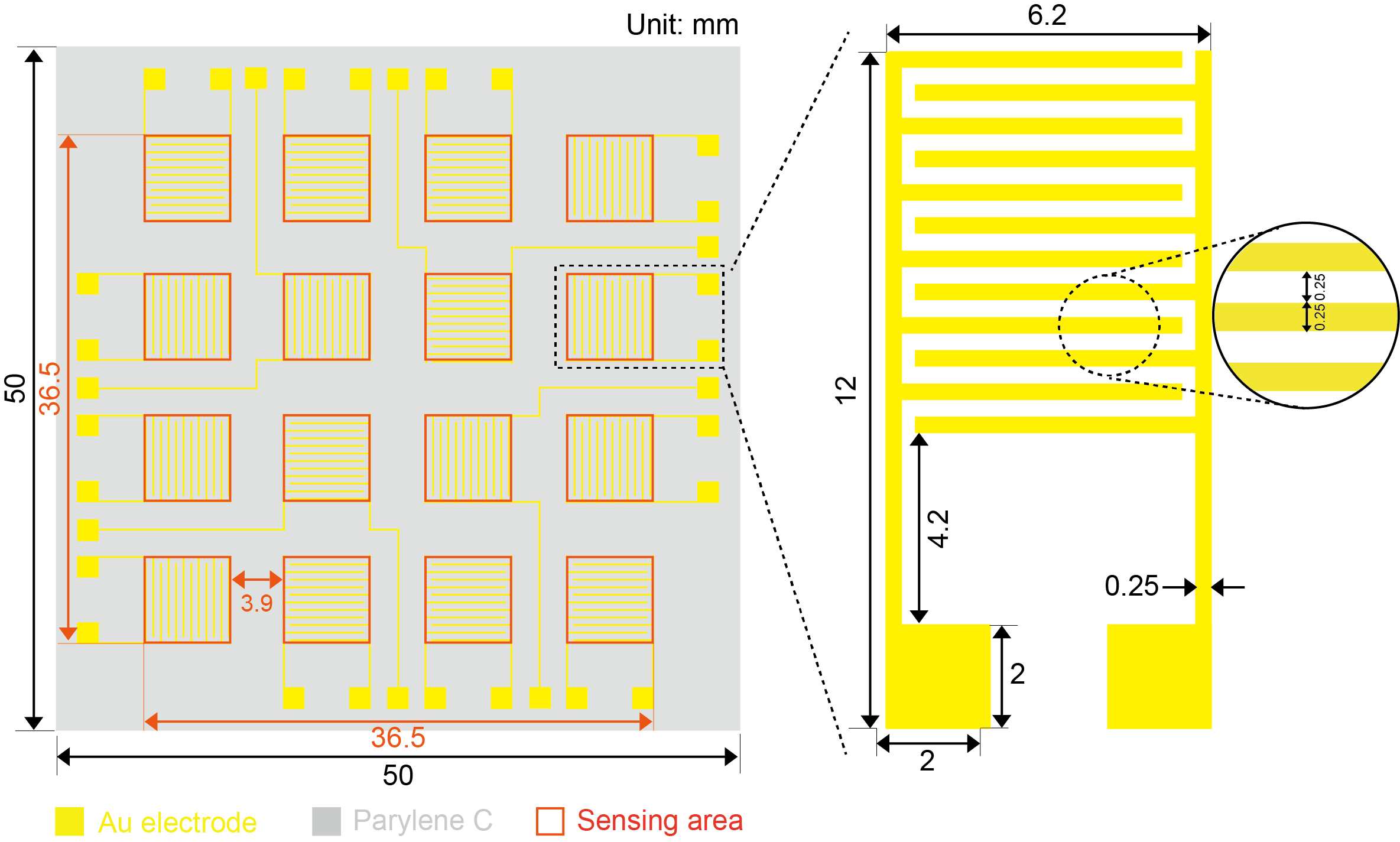


**Figure S4.** Schematic of wearable temperature sensor array.

**Table S1.** The calculated electrical conductivity of PEDOT:PSS composite film with different concentration of Ag flakes from 0 to 15 wt/%.

| Concentration of Ag flakes (%) | Electrical conductivity (S/cm) |
| --- | --- |
| 0 | 1355.3 |
| 3 | 1534.3 |
| 6 | 1626.8 |
| 9 | 1776.5 |
| 12 | 2038.4 |
| 15 | 1928.3 |


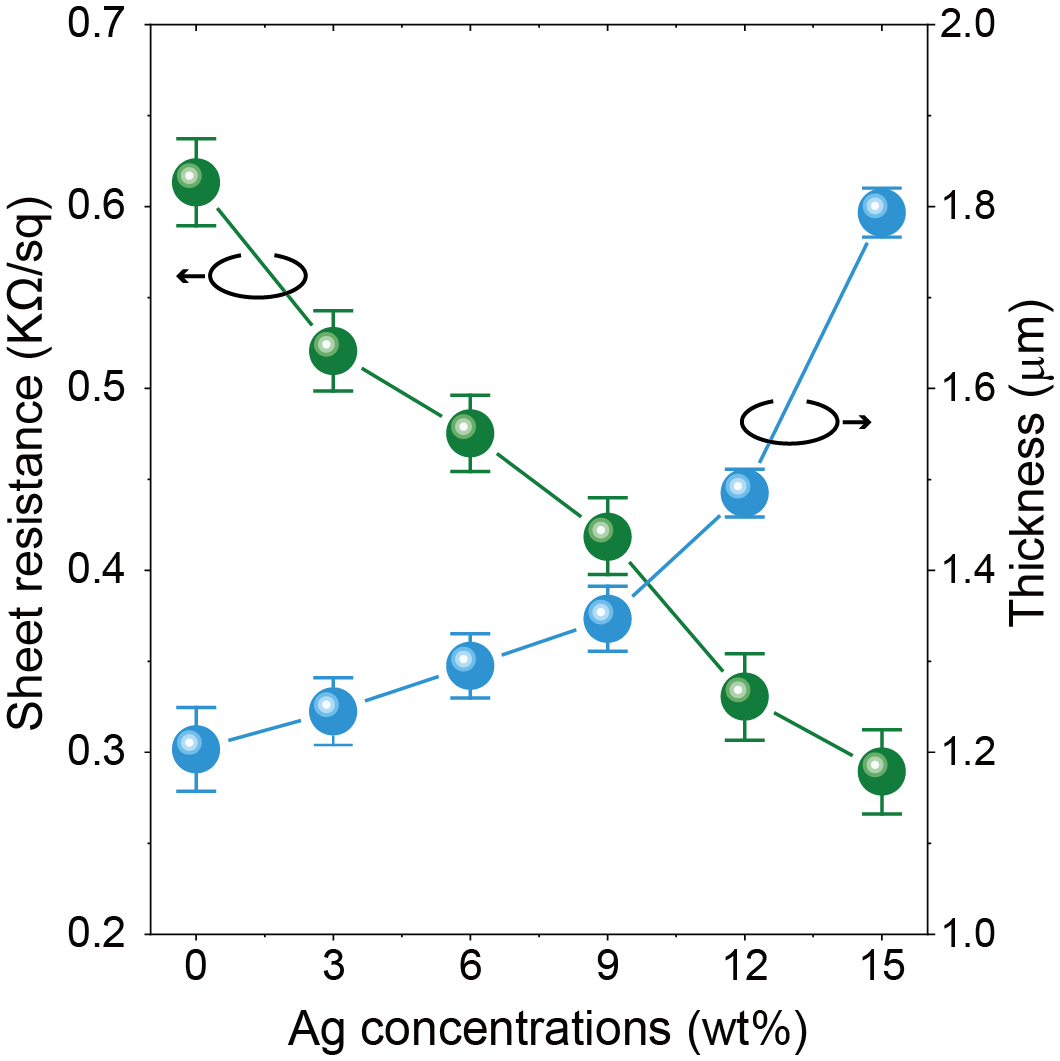


**Figure S5.** The measured sheet resistance and thickness of PEDOT:PSS composite film with different concentration of Ag flakes from 0 to 15 wt/%.

**
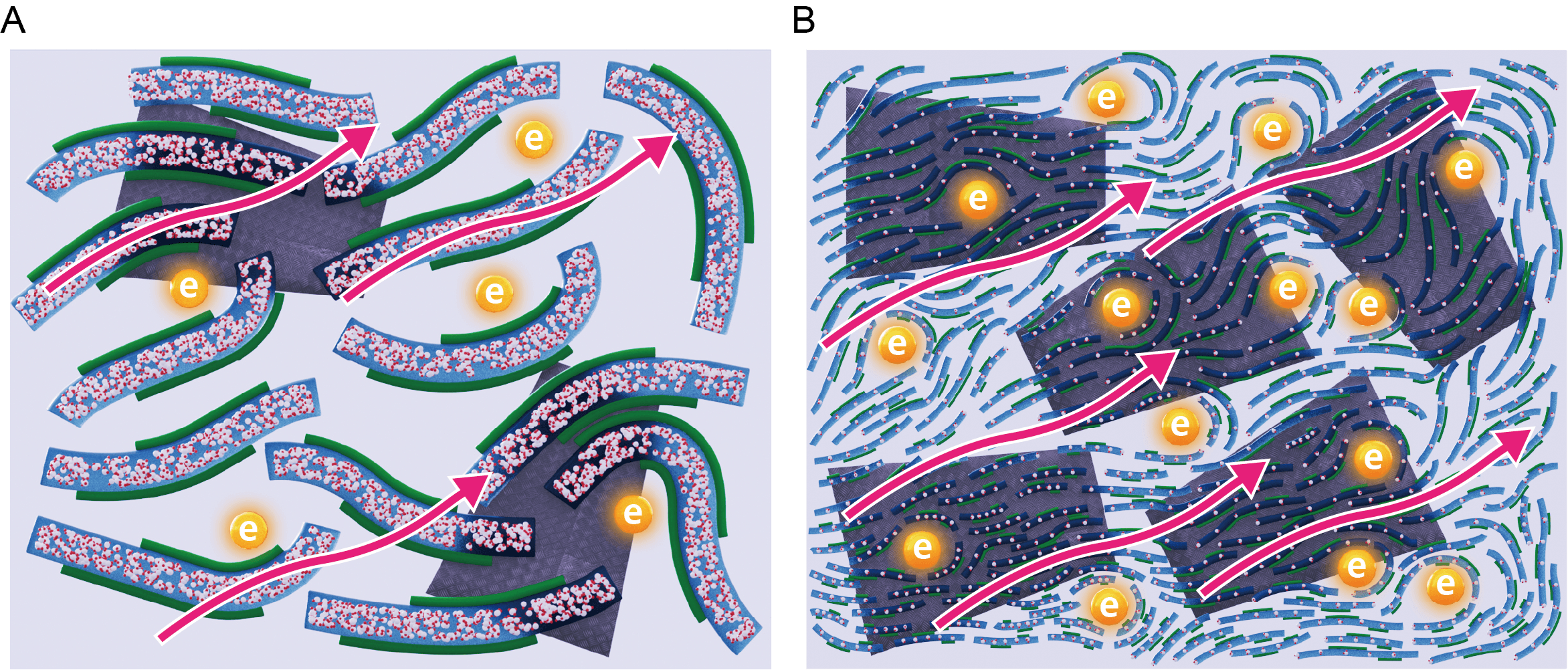
 Figure S6.** Illustration showing morphology change of PEDOT:PSS–Ag flake composite with the temperature of (A) 30 ℃ and (B) 50 ℃.


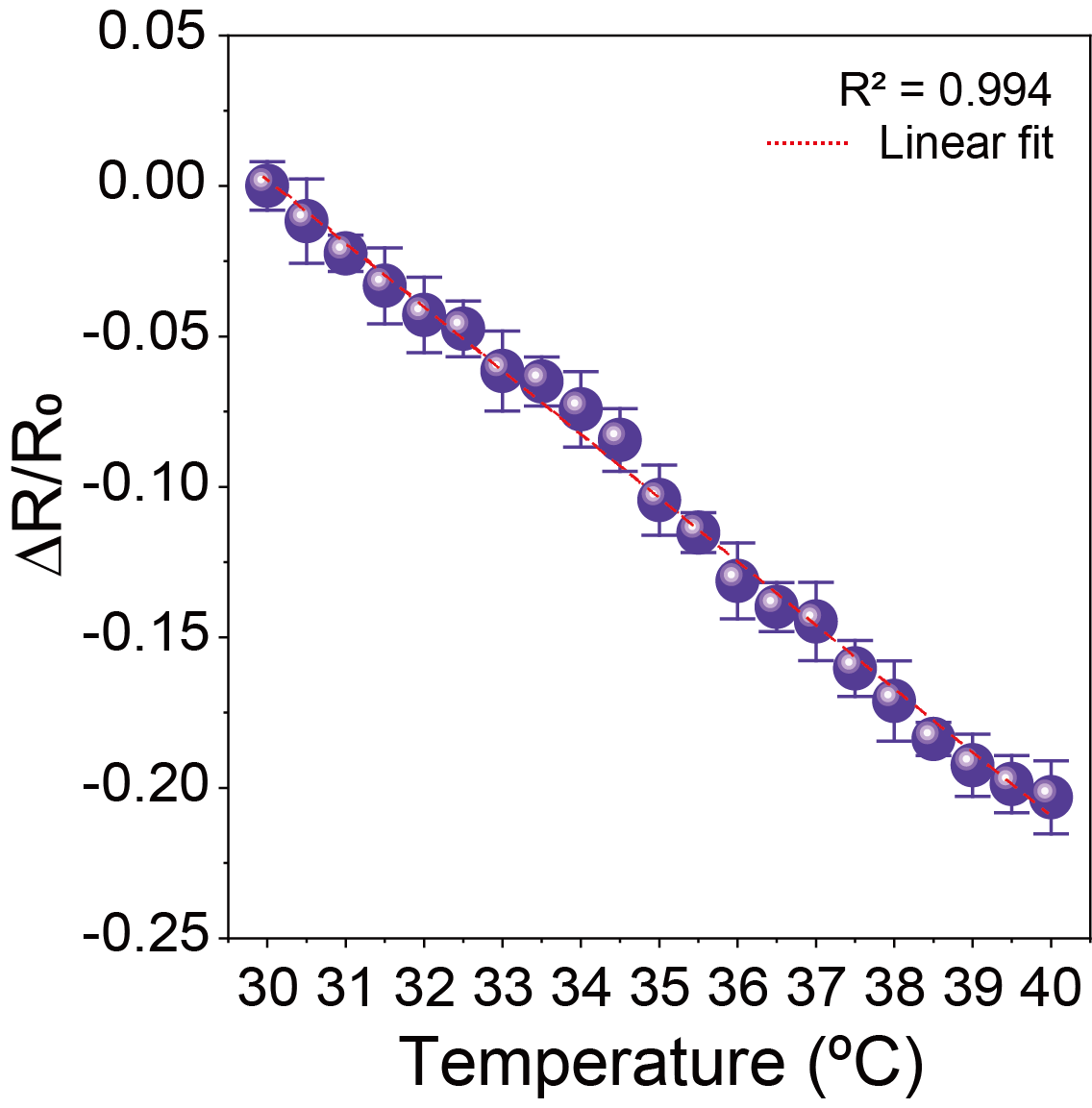


**Figure S7.** Relative resistance changes with temperature from 30 ℃ to 50 ℃ with resolution 0.5 ℃. The dashed red line indicates linear fitting from the experimental data. Error bars denote standard deviations measured from 30 devices.

**
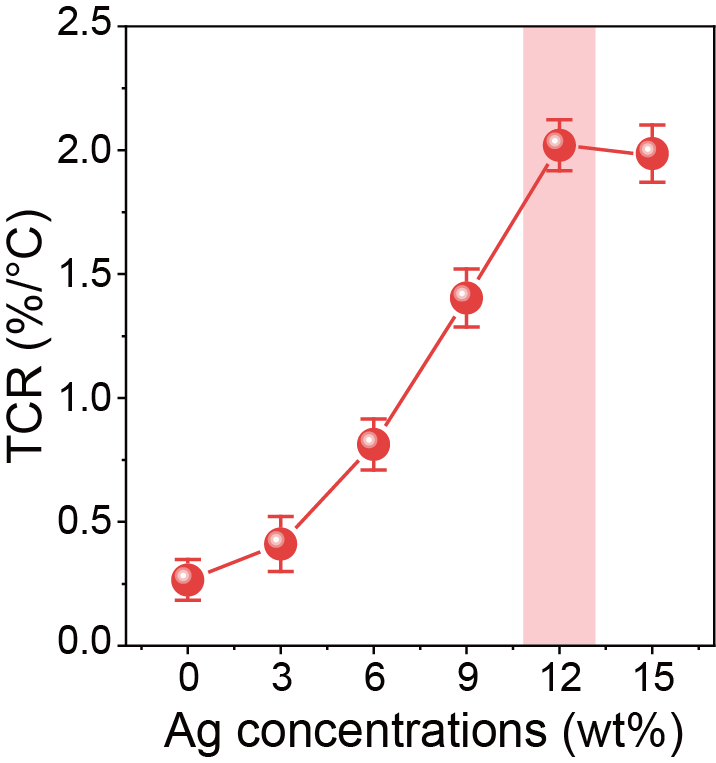
**

**Figure S8.** The trend of calculated temperature coefficient resistance (TCR) with different concentration of Ag flakes. The calculated TCR value was -2.02%/°C at 12% Ag flakes.


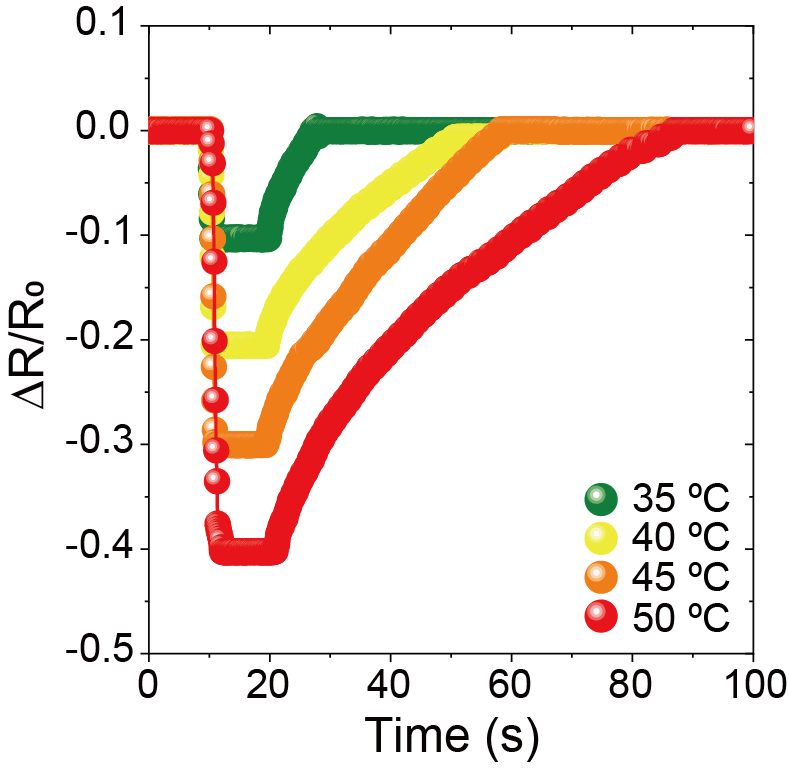


**Figure S9.** Real-time relative resistance changes of the prepared temperature sensors with different temperature change (Green: from 30 ℃ to 35 ℃ back to 30 ℃, yellow: from 30 ℃ to 40 ℃ back to 30 ℃, orange: from 30 ℃ to 45 ℃ back to 30 ℃, red: from 30 ℃ to 50 ℃ back to 30 ℃)

**Table S2.** Comparison of the parameters of the temperature sensor based on the material, measurement range, TCR, and response time.

| **Material** | **Measurement range (℃)** | **TCR**  **(%/℃)** | **Response time (s)** | **Ref.** |
| --- | --- | --- | --- | --- |
| Functionalized-rGO^a^ | 30 - 83 | 1.64 | 214 | ^[1]^ |
| rGO | 25 - 85 | 1.48 | 0.5 | ^[2]^ |
| MWCNT^b^ | 25 - 50 | 1.04 | 1.5 | ^[3]^ |
| MWCNT | 25 - 50 | 0.15 | 39 | ^[4]^ |
| GN^c^/PET^d^ | 25-100 | 0.83 | 13 | ^[5]^ |
| Carbon ink | 28-50 | 0.375 | 5 | ^[6]^ |
| AgNPs^e^ | -20 - 60 | 0.1713 | 33 | ^[7]^ |
| MWCNT/Graphene | 30-100 | 0.1373 | 340 | ^[8]^ |
| rGO/Aluminum | 35-45 | 0.06 | 20 | ^[9]^ |
| rGO | 25-45 | 1.30 | 0.443 | ^[10]^ |
| GNP^f^-PDMS^g^ | 25-120 | 0.214 | 1.6 | ^[11]^ |
| CNT/PET | 30-50 | 0.4 | 0.3 | ^[12]^ |
| rGO/Parlylene | 22-70 | 0.83 | 0.1 | ^[13]^ |
| rGO/PET^i^ | 30-100 | 0.6345 | 1.2 | ^[14]^ |
| PEDOT: PSS/Graphene | 35 - 45 | 0.064 | 20 | ^[15]^ |
| PEDOT:PSS/CNT^k^ | 22-48 | 0.61 | 18 | ^[16]^ |
| PEDOT:PSS/ FGO-PVDF^l^ | 10-30 | 0.395 | 40 | ^[17]^ |
| PEDOT:PSS/CNT | 20-80 | 0.64 | 4.8 | ^[18]^ |
| PEDOT: PSS/Carbon | 25-50 | 0.77 | 1.5 | ^[19]^ |
| PEDOT:PSS/GO | 25-100 | 1.09 | 18 | ^[20]^ |
| PEDOT:PSS/Ag flake | 30-50 | 2.02 | 0.41 | This work |

^a^rGO: Reduced graphene oxide

^b^MWCNT: Multi-Walled Carbon Nano Tube

^c^GN: Graphene nickel

^d^PET: polyethylene terephthalate

^e^AgNP: Ag nanoparticles

^f^GNP : Graphene Nanoplatelets

^g^PDMS: Polydimethylsiloxane

^h^PI: polyimide

^i^PET: Polyethylene terephthalate

^k^CNT: Carbon Nanotube

^l^FGO–PVDF: functionalized graphene oxide–polyvinylidene fluoride

**
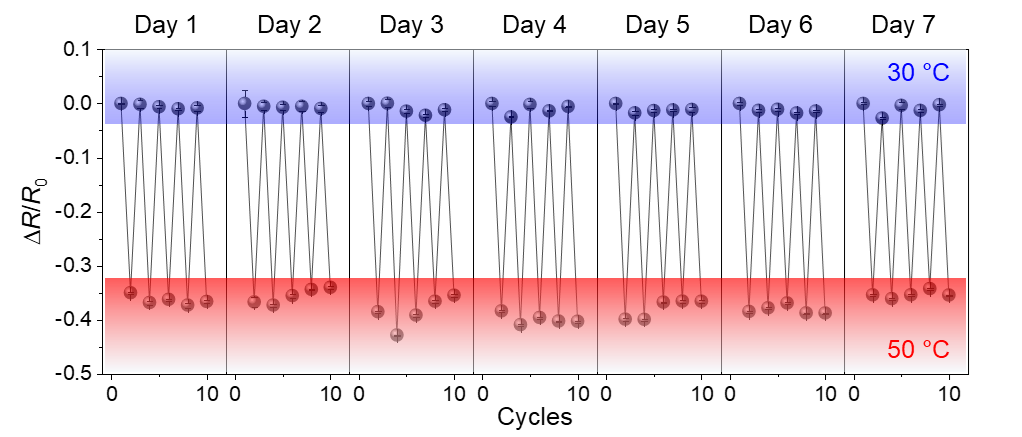
**

**Figure S10.** Relative resistance changes of the PEDOT:PSS–Ag flake temperature sensor measured at 30 °C and 50 °C over a period of 7 days (10 cycles per day).

**
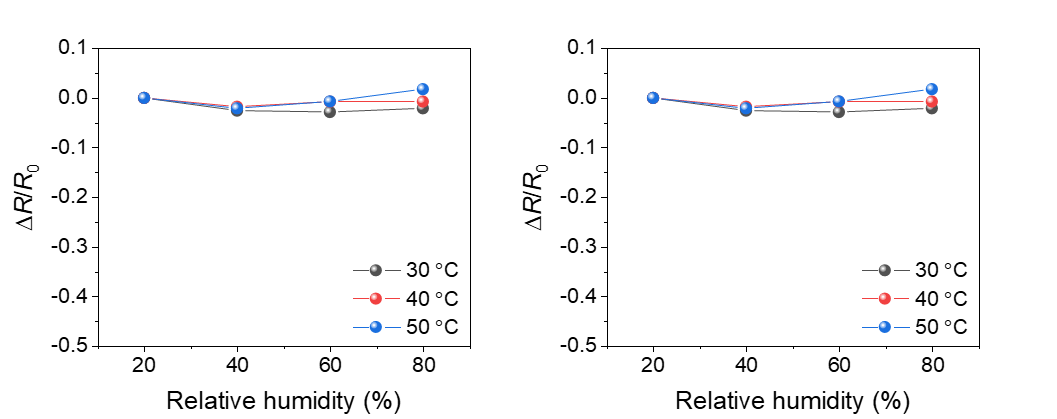
**

**Figure S11.** Relative resistance changes against relative humidity (RH) variations, where the temperatures were fixed at 30, 40, and 50 °C, respectively.

**
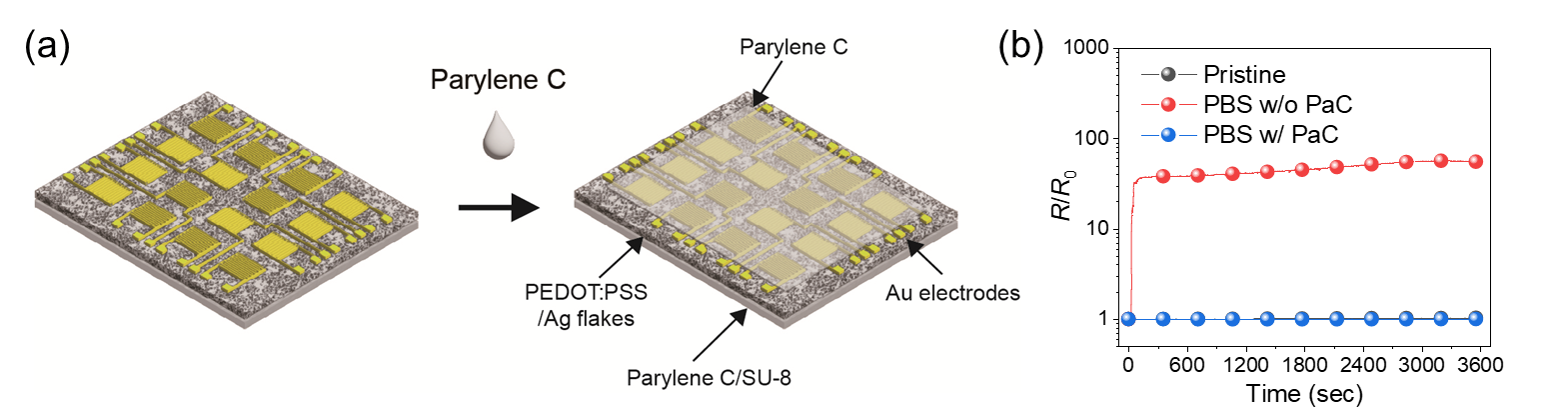
**

**Figure S12.** (a) Schematic of PaC passivation process for testing the droplet prevention. (b) Dynamics of relative resistance of the PEDOT:PSS–Ag flake composites at 30 °C under different conditions; (black) pristine condition without PaC passivation, (red) 100 μL PBS loading on the device without PaC passivation, and (blue) 100 μL PBS loading on the device with PaC passivation.


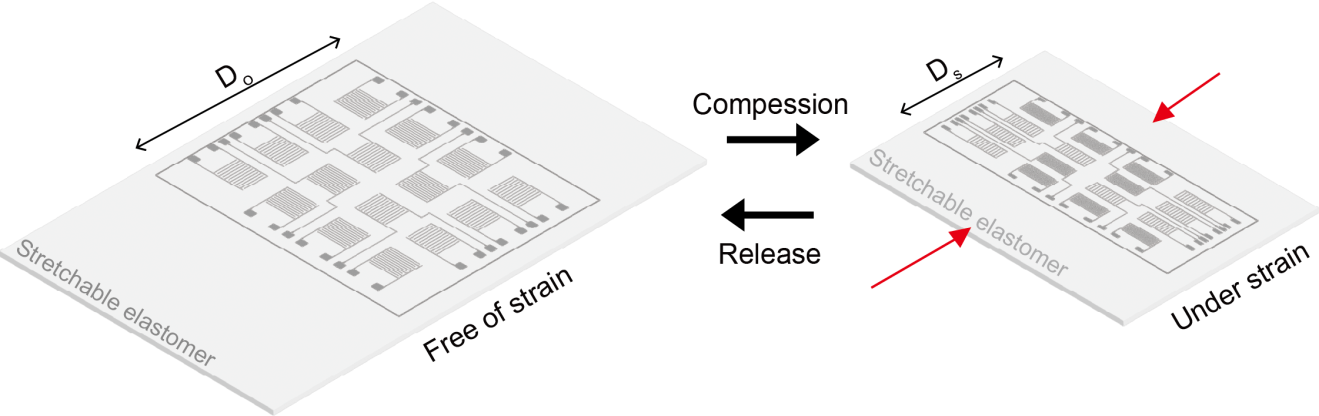


**Figure S13.** Schematic demonstrating compression-release testing of the temperature sensor on the stretchable elastomer.

**
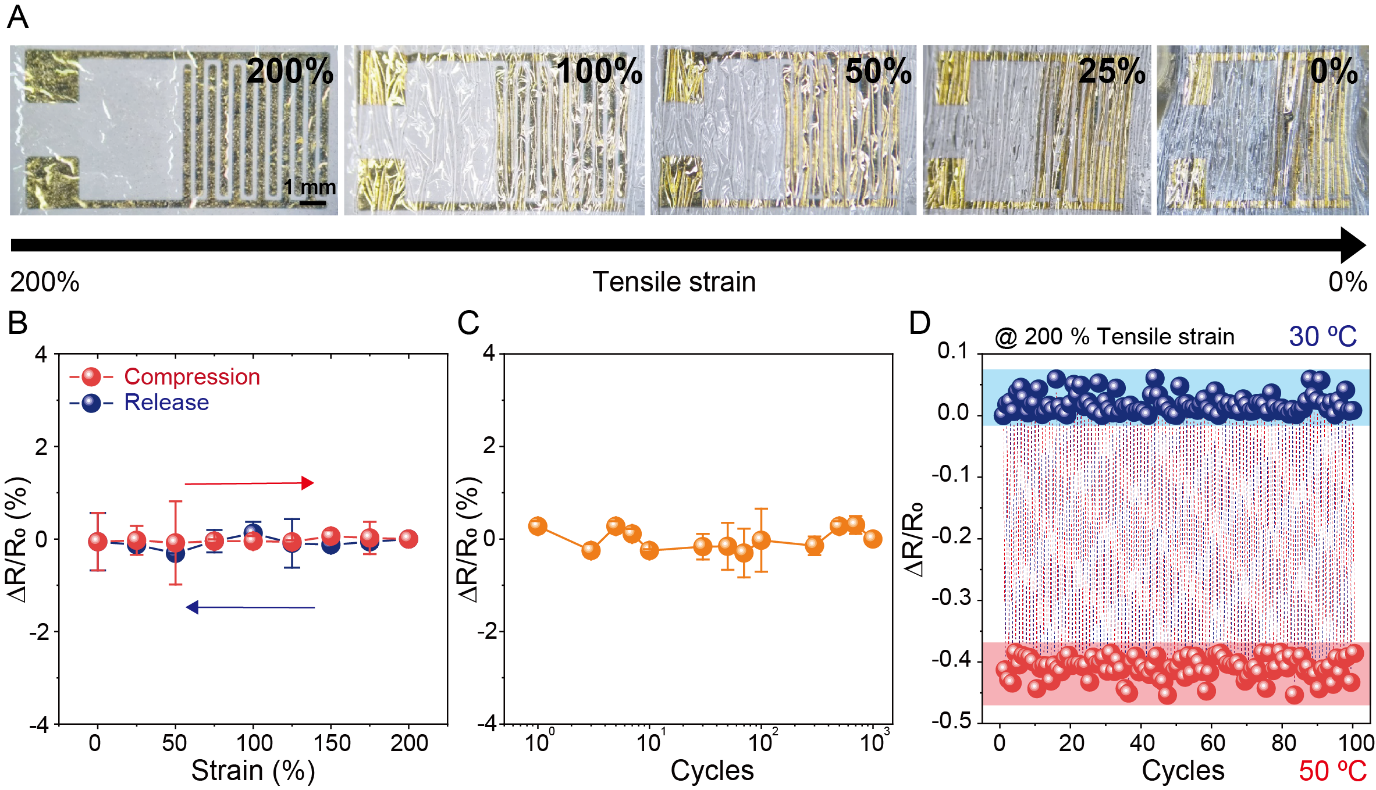
**

**Figure S14.** Morphology changes of temperature sensors under pre-stretched condition (200 % tensile strain) and compressed condition (0 % tensile strain) in vertical direction.


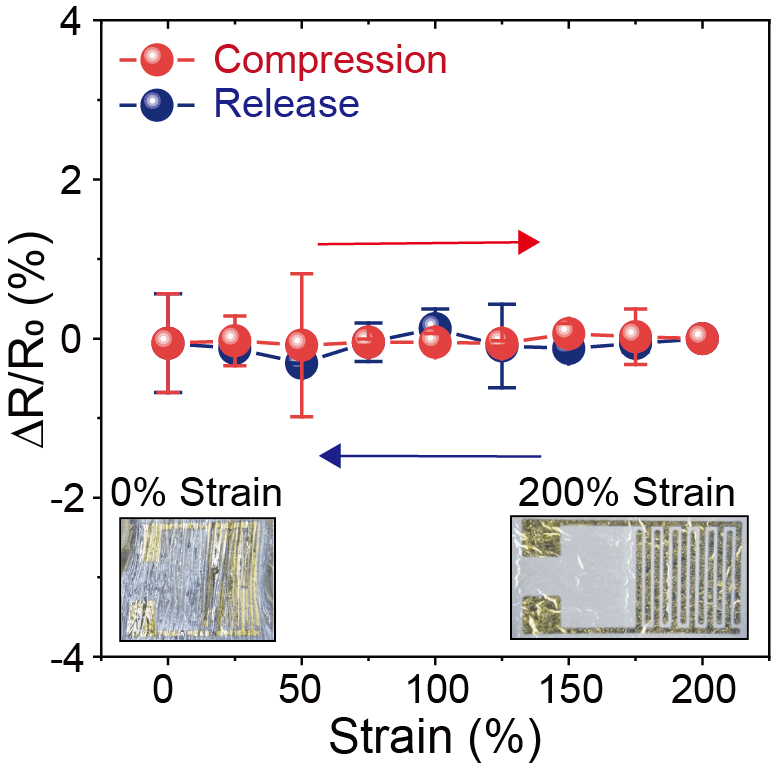


**Figure S15.** Relative resistance changes of the wearable temperature sensor from 0 to 200 % strain in vertical direction.

**
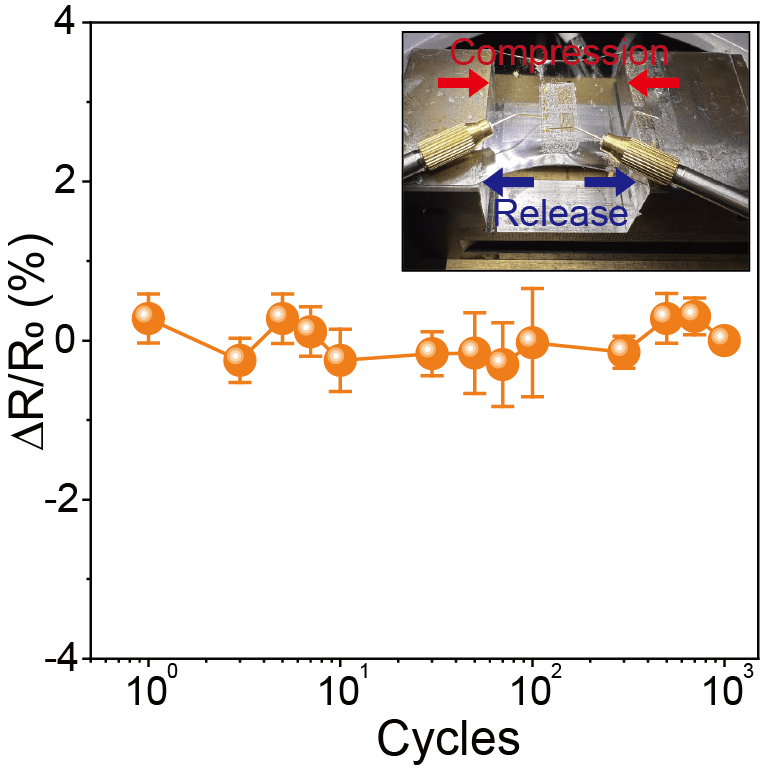
**

**Figure S16.** Relative resistance changes of the wearable temperature sensor repeated stretching from 0 % to 200 % strain over 1,000 cycles in vertical direction.


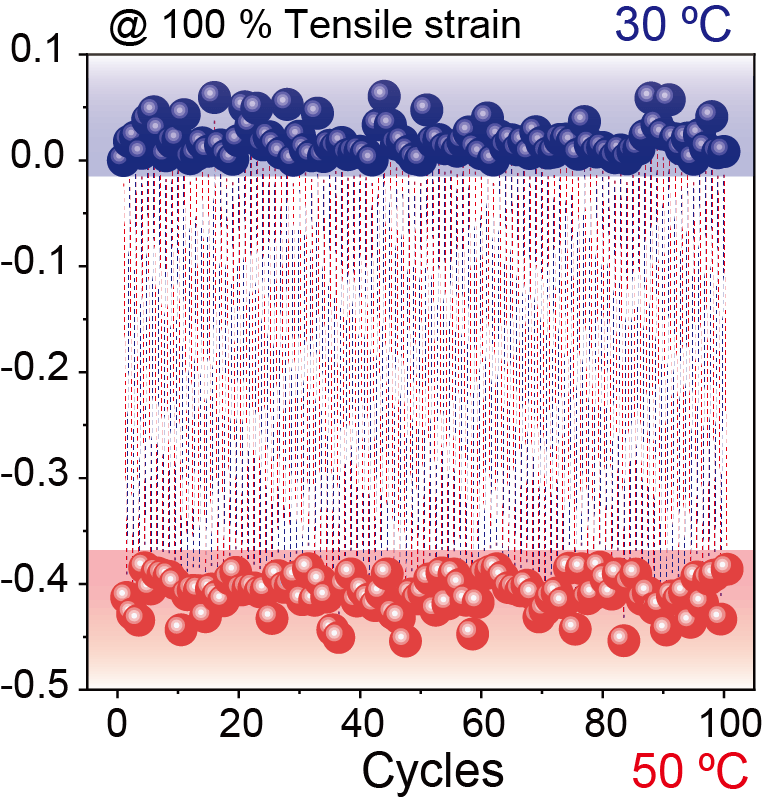


**Figure S17.** Repetitive measurement of the relative resistance changes of wearable temperature sensors switching between 30 ℃ and 50 ℃ for 100 cycles at 200 % tensile strain in vertical directions.

**Reference**

[1] D. Barmpakos, V. Belessi, R. Schelwald, G. Kaltsas, *Nanomaterials* **2021**, *11*, 2025.

[2] D. Kong, L. T. Le, Y. Li, J. L. Zunino, W. Lee, *Langmuir* **2012**, *28*, 13467.

[3] B. A. Kuzubasoglu, E. Sayar, C. Cochrane, V. Koncar, S. K. Bahadir, *J Mater Sci: Mater Electron* **2021**, *32*, 4784.

[4] B. A. Kuzubasoglu, E. Sayar, S. K. Bahadir, *IEEE Sensors J.* **2021**, *21*, 13090.

[5] M. Hilal, J. I. Han, *IEEE Sensors J.* **2020**, *20*, 5146.

[6] S. Ali, S. Khan, A. Bermak, *IEEE Access* **2019**, *7*, 163981.

[7] J. Zikulnig, M. Khalifa, L. Rauter, H. Lammer, J. Kosel, *Chemosensors* **2021**, *9*, 95.

[8] J. Huang, X. Yang, S.-C. Her, Y.-M. Liang, *Sensors* **2019**, *19*, 317.

[9] P. Sehrawat, Abid, S. S. Islam, P. Mishra, *Sensors and Actuators B: Chemical* **2018**, *258*, 424.

[10] Q. Liu, H. Tai, Z. Yuan, Y. Zhou, Y. Su, Y. Jiang, *Advanced Materials Technologies* **2019**, *4*, 1800594.

[11] J. Yang, D. Wei, L. Tang, X. Song, W. Luo, J. Chu, T. Gao, H. Shi, C. Du, *RSC Adv.* **2015**, *5*, 25609.

[12] V. S. Turkani, D. Maddipatla, B. B. Narakathu, B. J. Bazuin, M. Z. Atashbar, *Sensors and Actuators A: Physical* **2018**, *279*, 1.

[13] L. Wu, J. Qian, J. Peng, K. Wang, Z. Liu, T. Ma, Y. Zhou, G. Wang, S. Ye, *J Mater Sci: Mater Electron* **2019**, *30*, 9593.

[14] W. Honda, S. Harada, T. Arie, S. Akita, K. Takei, *Advanced Functional Materials* **2014**, *24*, 3299.

[15] T. Vuorinen, J. Niittynen, T. Kankkunen, T. M. Kraft, M. Mäntysalo, *Sci Rep* **2016**, *6*, 35289.

[16] R. Polanský, R. Soukup, J. Řeboun, J. Kalčík, D. Moravcová, L. Kupka, M. Švantner, P. Honnerová, A. Hamáček, *Sensors and Actuators A: Physical* **2017**, *265*, 111.

[17] B. B. Maskey, K. Shrestha, J. Sun, H. Park, J. Park, S. Parajuli, S. Shrestha, Y. Jung, S. Ramasundaram, G. R. Koirala, G. Cho, *RSC Adv.* **2020**, *10*, 12407.

[18] O. Ozioko, Y. Kumaresan, R. Dahiya, in *2020 IEEE International Conference on Flexible and Printable Sensors and Systems (FLEPS)*, **2020**, pp. 1–4.

[19] C. Bali, A. Brandlmaier, A. Ganster, O. Raab, J. Zapf, A. Hübler, *Materials Today: Proceedings* **2016**, *3*, 739.

[20] Z. Cui, F. R. Poblete, Y. Zhu, *ACS Appl. Mater. Interfaces* **2019**, *11*, 17836.
